# Immune-adaptive pathogen variation reveals targetable mediators of gram-positive bacterial killing in macrophages

**DOI:** 10.1126/sciadv.aea0375

**Published:** 2026-02-27

**Authors:** Clark D. Russell, Jennifer Marshall, Brian J. McHugh, Bartosz J. Michno, Justyna Cholewa-Waclaw, Gonzalo Yebra, Jelimo Chepsat, Gareth-Rhys Jones, Martin P. McHugh, Nicola N. Lynskey, Stephen A. Renshaw, Tomasz K. Prajsnar, J. Kenneth Baillie, J. Ross Fitzgerald, David H. Dockrell

**Affiliations:** ^1^ University of Edinburgh Centre for Inflammation Research, Institute for Regeneration and Repair, Edinburgh EH16 4UU, UK.; ^2^ Baillie-Gifford Pandemic Science Hub, Institute for Regeneration and Repair, The University of Edinburgh, Edinburgh EH16 4UU, UK.; ^3^ Department of Evolutionary Immunology, Institute of Zoology and Biomedical Research, Faculty of Biology, Jagiellonian University, Kraków 31-007, Poland.; ^4^ Doctoral School of Exact and Natural Sciences, Jagiellonian University, Kraków 31-007, Poland.; ^5^ High Content Screening Facility, Institute for Regeneration and Repair, The University of Edinburgh, Edinburgh EH16 4UU, UK.; ^6^ Roslin Institute, The University of Edinburgh, Midlothian EH25 9RG, UK.; ^7^ School of Infection and Immunity, University of Glasgow, Glasgow G12 8TA, UK.; ^8^ Department of Clinical Microbiology, Royal Infirmary of Edinburgh, Edinburgh EH16 4SA, UK.; ^9^ School of Medicine, University of St Andrews, St Andrews KY16 9TF, UK.; ^10^ Florey Institute, Bateson Centre and Division of Clinical Medicine, School of Medicine and Population Health, Sheffield S10 2RX, UK.

## Abstract

Host-directed therapies for bacterial infections can provide an adjunct or alternative to conventional antimicrobials, mitigating the impact of antimicrobial resistance. However, therapeutically targetable mediators of innate immune bacterial killing remain elusive. We hypothesized that immune-adaptive pathogen evolution could provide an informative perspective on this problem. We examined the interaction of a genetically diverging hypervirulent *Streptococcus pneumoniae* (pneumococcus) serotype with macrophages, identifying closely phylogenetically related isolates with differential susceptibility to intracellular killing. We reasoned that macrophage genes relatively suppressed during pathogen escape from killing were likely to encode mediators normally promoting bacterial killing. This led to the validation of ACOD1 and its product itaconate, NAMPT, and P2RX7 as host defense factors against pneumococci and related gram-positive pathogens. Last, we repurposed the antihistamine clemastine to augment phagolysosomal bacterial killing, via P2RX7, as a candidate host-directed therapy against pneumococci and vancomycin-resistant *Enterococcus faecium*. Overall, we show that pathogen-centric host screening can aid identification of microbicidal responses as targets for host-directed therapies.

## Teaser

Immune-adaptive pathogen variation is a tool to identify mediators of macrophage bacterial killing and therapeutic opportunities.

## INTRODUCTION

The global spread of antimicrobial resistance threatens the efficacy of antimicrobial chemotherapy (*1*). Augmenting host microbicidal responses represents an alternative therapeutic strategy (host-directed therapy) that can reduce our reliance on antimicrobials and mitigate the impacts of antimicrobial resistance (*2*). However, identifying specific targetable mediators of antibacterial innate immune responses represents a major challenge due to the complexity and heterogeneity of microbicidal responses in vivo. In the current study, we addressed this challenge by adopting a pathogen-centric perspective on the host response: We reasoned that microbicidal responses which a successful pathogen variant has evolved to escape will be critical to host defense.

Immune responses exert demonstrable selective pressure on pathogen evolution that can result in escape from otherwise effective host responses (*3*). Examples include host species-specific variation of the *Staphylococcus aureus* superantigen staphylococcal enterotoxin-like toxin X associated with differences in T cell receptor activation (*4*) and adaptive mutation of *Legionella pneumophila* (*5*) and *S. aureus* (*6*) during serial passage in macrophages in vitro resulting in enhanced intramacrophagic survival. The capacity of genetic diversity underpins the adaptability of bacterial pathogens under selective pressure from host immune responses (*3*) so immune-adaptive variants can aid identification of critical elements of the host response exerting selective pressure. Serotype 1 *Streptococcus pneumoniae* (pneumococci) causes disease that is clinically and epidemiologically distinct from other capsular serotypes of pneumococci and represents putative adaptive variants (*7*). These strains have an unusually high attack rate (*8*, *9*), behaving as primary pathogens causing invasive disease in otherwise healthy hosts including at extrapulmonary sites (*7*), and are associated with disease outbreaks and epidemics (*10*). These hypervirulent strains exhibit genetic variation within the same capsular background and are undergoing diversifying selection in geographically discrete lineages as distinct pathotypes (*7*, *11*). We aimed to use insights from host adaptation in these strains to highlight microbicidal responses that they have escaped from but that usually contribute to effective pathogen control.

Macrophage intracellular bacterial killing is a key bottleneck in host defense against bacteria, including pneumococci, and was the focus of this study. Depletion of alveolar macrophages in mice reduces resistance to pneumococcal pneumonia (*12*), while occupational exposure to welding fumes leads to alveolar macrophage dysfunction in humans, associated with pneumococcal pneumonia and invasive disease (*13*–*15*). Furthermore, pneumococcal intracellular persistence within a subset of splenic macrophages provides a reservoir for bacteraemia (*16*), and humans with defective macrophage intracellular killing of pneumococci due to chronic obstructive pulmonary disease (COPD) or HIV-1 infection are at increased risk of pneumococcal pneumonia and invasive disease (*17*, *18*).

Here, we probed macrophage bacterial killing mechanisms using hypervirulent serotype 1 pneumococcal clinical isolates. This approach led to the identification of distinct macrophage transcriptional responses underpinning the differential ability to kill closely related bacterial isolates. We found that aconitate decarboxylase 1 (ACOD1) and its product itaconate, nicotinamide phosphoribosyltransferase (NAMPT), and purinergic receptor P2X7 (P2RX7) mediate macrophage intracellular bacterial killing in vitro and pathogen clearance in vivo. Furthermore, we identified that clemastine, which potentiates P2RX7 signaling (*19*), represents a candidate for drug repurposing as a host-directed therapy for gram-positive bacterial infections. Our results demonstrate the underappreciated potential of a pathogen-centric screening approach to reveal critical host microbicidal responses.

## RESULTS

### Identification of pneumococcal variants escaping macrophage intracellular killing

**Fig. 1.**
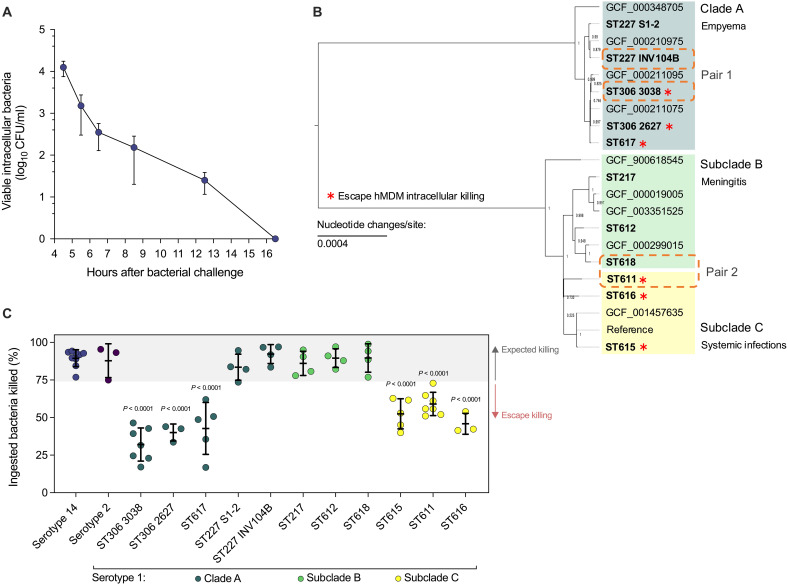
Screening *S. pneumoniae* serotype 1 isolates for escape from macrophage intracellular killing. (**A**) Kinetics of hMDM intracellular killing of serotype 14 *S. pneumoniae* multiplicity of infection (MOI) of 10 (*n* = 6 biological replicates). (**B**) Maximum-likelihood phylogenetic tree of serotype 1 *S. pneumoniae* study isolates. Numbers at nodes represent bootstrap values (1 = highest confidence in position of node). Scale bar indicates number of nucleotide changes per site. (**C**) Susceptibility of *S. pneumoniae* isolates to early intracellular killing by hMDMs (*n* = 3 to 9 biological replicates, shown as individual data points). Gray shading indicates expected killing. Serotype 1 means were compared to serotype 14 by analysis of variance (ANOVA) with Dunnett’s multiple comparisons test. [(A) and (C)] Data presented as means and SD.

To determine the kinetics of intracellular killing of pneumococci by macrophages, we first challenged human monocyte-derived macrophages (hMDMs) from healthy donors with opsonized serotype 14 pneumococci. Serotype 14 was chosen because of its intermediate invasive disease potential relative to serotype 1 (*8*, *9*) and association with opportunistic pathogenesis in people with underlying comorbidity (*9*), making it broadly representative of invasive pneumococcal isolates. We observed an early phase of extensive killing (Fig. 1A), consistent with effective phagolysosomal killing (*20*), followed by later clearance of remaining viable intracellular bacteria, which is known to be mediated by apoptosis-associated killing mechanisms (*21*). As most ingested bacteria were cleared in the early phase of killing, this time point was chosen to screen 11 clinical isolates of serotype 1 pneumococci recovered from humans with invasive pneumococcal disease (table S1) (*11*).

To infer the evolutionary relationship of the study isolates to the pneumococcal population as a whole, we used 128 complete pneumococcal genome assemblies to produce a core genome-based phylogeny. This demonstrated that all serotype 1 genomes clustered together in a discrete clade (fig. S1A). After stripping recombinant regions, a maximum-likelihood phylogenetic tree of the study isolates revealed two major clades with a further two subclades (Fig. 1B). The clades correlated with previously described geographic lineages and pathotypes of serotype 1 pneumococci (Fig. 1B) (*11*). After adjusting the multiplicity of infection for each study isolate to achieve an equivalent initial ingested load of bacteria (fig. S1B), we quantified early intracellular bacterial killing by hMDM, identifying differences in susceptibility to killing within clade A and between subclades B and C (Fig. 1C). We observed that the resistant isolates within clade A belonged to sequence types ST306 and ST617, carrying pneumolysin allele 5, which is nonhemolytic and associated with failure to activate the NLRP3 (NOD-, LRR-, and pyrin domain-containing protein 3) inflammasome resulting in lack of caspase 1 activation and subsequent interleukin-1β (IL-1β) release (*22*–*24*). To determine whether production of nonhemolytic pneumolysin alone, independently of other genetic variation present in the study isolates, could reproduce the observed resistance to killing in an otherwise susceptible isolate, we examined a D39 allelic exchange mutant expressing allele 5 pneumolysin. A D39 mutant was used because of the technical constraints genetically modifying serotype 1 isolates (*7*). hMDM early killing of this mutant was unaltered compared to wild-type D39. Early bacterial killing was also unaltered by treatment with a selective irreversible caspase 1 inhibitor and recombinant human IL-1 receptor antagonist, providing further evidence that NLRP3 inflammasome evasion by nonhemolytic pneumolysin variants was not the basis for reduced early killing (fig. S1, C and D). Together, these findings indicate the observed escape of clade A isolates from hMDM early killing is not a consequence of the known immune evasion mechanism related to nonhemolytic pneumolysin and lack of NLRP3 inflammasome activation, suggesting that additional adaptations to other host responses exist in these serotype 1 isolates. Detection of experimentally verified virulence genes from the bacterial genome sequences was performed using the Virulence Factors of Pathogenic Bacteria database (*25*) to determine whether presence/absence correlated with resistance to macrophage killing; however, no correlations were observed (table S1).

We chose the resistant isolates ST306 3038 (clade A; nonhemolytic pneumolysin) and ST611 (subclade C; hemolytic pneumolysin) for further investigation as isolates with evidence of adaptation to escape macrophage intracellular killing. We then used the phylogenetic analysis of the serotype 1 study isolates to identify the most closely related isolates that lacked resistance to intracellular killing for use as comparators: We paired ST227 INV104B (clade A) with ST306 3038 (pair 1) and ST618 (subclade B, with a shared common ancestor with ST611) with ST611 (pair 2; Fig. 1B). Together, we have identified pairs of closely related clinical isolates of serotype 1 pneumococci with differences in susceptibility to macrophage intracellular killing to use as tools to investigate the responses underpinning successful killing.

### Macrophage transcriptomic programs associated with differential intracellular bacterial killing

**Fig. 2.**
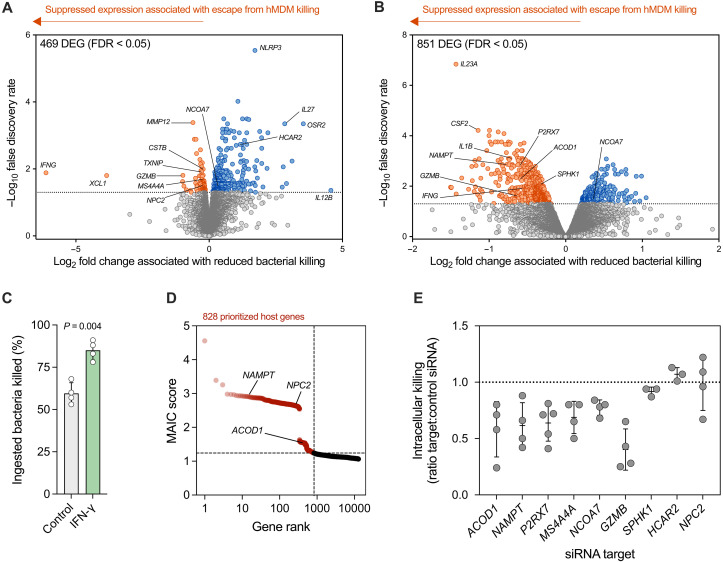
Macrophage transcriptional profiles associated with differential intracellular killing identify host defense factors. Volcano plots of log_2_ fold change difference in hMDM gene expression 4 hours after bacterial challenge for (**A**) pair 1 (*S. pneumoniae* ST306 3038 versus ST227 INV104B) and (**B**) pair 2 (*S. pneumoniae* ST611 versus ST618); *n* = 5 biological replicates for each comparison. Horizontal dotted lines indicated false discovery rate (FDR) of 0.05 (bottom) and 0.01 (top). Data points (genes) are colored on the basis of differential expression (FDR < 0.05): increased (blue), decreased (orange), or no difference (gray). (**C**) Effect of recombinant human IFN-γ pretreatment (50 ng/ml) on hMDM early intracellular killing of *S. pneumoniae* ST611 MOI of 5 (*n* = 4 biological replicates), compared using paired *t* test. (**D**) Distribution of meta-analysis by information content (MAIC) scores and ranks. Red shading indicates prioritized genes determined using the unit invariant knee method. (**E**) Effect of small interfering RNA (siRNA) knockdown on hMDM early intracellular killing of *S. pneumoniae* ST618 MOI of 10 (*n* = 3 to 5 biological replicates). A ratio of 1.0 (dotted line) between target:control siRNA indicates no difference in killing, whereas a ratio of < 1.0 indicates a reduction in killing when hMDMs are transfected with the targeting siRNA relative to nontargeting control. [(C) and (E)] Data points represent biological replicates.

To understand the host transcriptional responses underpinning the differential killing of these isolates, we then performed bulk RNA sequencing (RNA-seq) on hMDMs 4 hours after bacterial challenge (Fig. 2, A and B). We challenged macrophages from each independent donor (*n* = 5) separately with each isolate from either pair 1 or pair 2 in tandem, using a paired design (fig. S2). We performed differential gene expression analysis to identify hMDM genes with differing expression levels between the isolates in each pair. We reasoned that host genes that were activated in successful killing, but not during pathogen escape from killing, were likely to encode core elements of the macrophage microbicidal response. We therefore focused on genes that were relatively suppressed during interaction with the more resistant pathogen variant, indicating that escape from these responses could be causal in the reduced capacity for bacterial killing. These included several genes with established roles in host defense and phagolysosome activity specifically, including matrix metalloproteinase 12 (*MMP12*) (*26*), cystatin B (*CSTB*) (*27*), thioredoxin-interacting protein (*TXNIP*) (*28*), IL-1β (*IL1B*), interferon-γ (IFN-γ; *IFNG*; suppressed in both pairs), and colony-stimulating factor 2 (*CSF2*) (*29*). Gene set enrichment analysis of differentially expressed genes identified terms related to lipid metabolism, IFN-γ, and several classical intramacrophagic pathogens (*Mycobacterium tuberculosis*, *Leishmania* spp., *Legionella* spp., and *Salmonella* spp.; fig. S3, A to D). As proof of principle that suppressed genes could be causally linked to reduced bacterial killing, we pretreated hMDM with IFN-γ, resulting in increased early killing of the resistant isolate ST611 (Fig. 2C).

We used meta-analysis by information content (MAIC) as one approach to prioritize differentially expressed genes for further investigation. The MAIC algorithm is a previously described and validated computational approach to aggregate gene lists and to prioritize results (*30*, *31*). In addition to the gene lists generated in the current study, we included 45 lists from 29 published diverse genome-scale studies of host responses to pneumococci (fig. S3, E to G). The MAIC analysis identified 828 host factors with the strongest cumulative evidence for a role in the host response to pneumococci, including *NAMPT*, *ACOD1*, and *NPC2* (Fig. 2D).

Ultimately, nine genes of interest were selected for further investigation, on the basis of MAIC, differential expression in both pairs, membership of enriched pathways, and insight from classical intramacrophagic pathogens highlighted by gene set enrichment (fig. S3, A to H). We then subjected these genes of interest to small interfering RNA (siRNA) knockdown in hMDMs, confirming that loss of *NAMPT*, *ACOD1*, *P2RX7*, *GZMB*, *NCOA7*, or *MS4A4A* was associated with reduced early intracellular bacterial killing, whereas loss of *NPC2*, *HCAR2*, and *SPHK1* was not (Fig. 2E). Together, differential macrophage transcriptional responses to immune-adaptive pathogen variants led to the identification and validation of mediators of bacterial killing.

### The ACOD1 product itaconate is a microbicidal effector against gram-positive bacteria

**Fig. 3.**
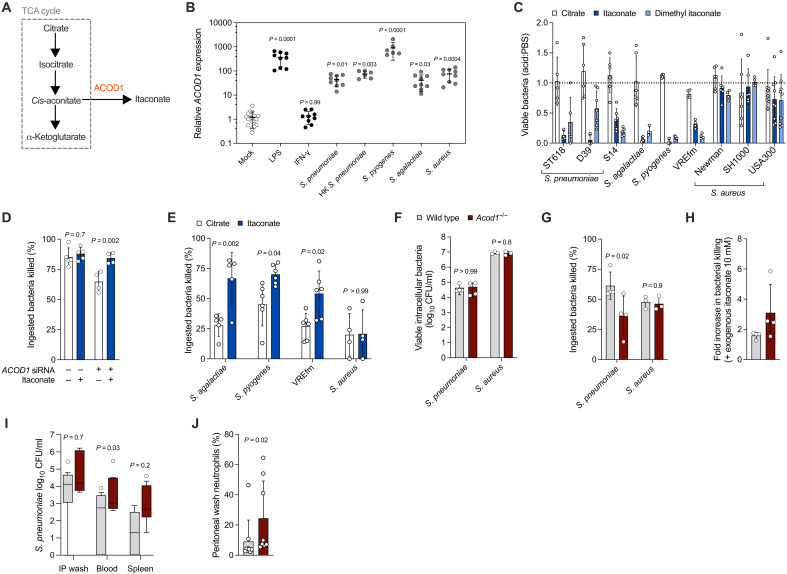
ACOD1 and itaconate contribute to macrophage host defense against gram-positive bacteria. (**A**) Schematic representation of itaconate production. (**B**) hMDMs were either mock treated or challenged for 4 hours with MOI of 10 *S. pneumoniae* ST618 (live or heat killed, “HK”), *S. agalactiae* ST23, *S. pyogenes* M1T1, or *S. aureus* Newman; LPS (100 ng/ml); or IFN-γ (50 ng/ml) (*n* = 9 biological replicates). Gene expression relative to *GAPDH* was calculated using the delta-delta Ct (cycle threshold) method. Conditions were compared to mock treatment using ANOVA with Dunn’s multiple comparisons test. (**C**) Microbicidal effect of 5 mM itaconate, dimethyl itaconate, or citrate against planktonic gram-positive bacteria (*n* = 6 to 9 technical replicates). The *S. agalactiae* strain was ST23. (**D**) Effect of *ACOD1* siRNA knockdown and 10 mM exogenous itaconate on hMDM early intracellular killing of *S. pneumoniae* ST618 MOI of 10 (*n* = 4 to 5 biological replicates), compared using ANOVA with Šidák’s multiple comparisons test. Citrate (10 mM) was used as the control for itaconate. (**E**) Effect of 10 mM exogenous itaconate compared to citrate on hMDM early intracellular killing of gram-positive bacteria MOI of 5 (*n* = 4 to 6 biological replicates), compared using ANOVA with Šidák’s multiple comparisons test. The *S. agalactiae* strain was ST23 and *S. aureus* was Newman. (**F**) Ingested bacteria and (**G**) early intracellular killing (*S. pneumoniae* ST618 MOI of 10 or *S. aureus* Newman MOI of 5) for mBMDMs from wild-type and *Acod1*^−/−^ mice (*n* = 3 to 4 biological replicates), compared using ANOVA with Šidák’s multiple comparisons test. (**H**) Effect of 10 mM exogenous itaconate on wild-type and *Acod1*^−/−^ mBMDM early intracellular killing of *S. pneumoniae* ST618 MOI of 10. Comparison of wild-type and *Acod1^−/−^* mouse (**I**) bacterial clearance and (**J**) peritoneal wash neutrophils, 24 hours after intraperitoneal infection with 10^2^ CFU *S. pneumoniae* ST227 INV104B (*n* = 9 male mice per group). (I) compared using Kruskal-Wallis test with Dunn’s multiple comparisons test; (J) compared using Mann Whitney test. [(B) to (J)] Data presented as means and SD; data points represent biological replicates. IP, intraperitoneal.

ACOD1, which catalyzes itaconate production from cis-aconitate (Fig. 3A), gene expression is up-regulated in macrophages infected with *M. tuberculosis* (*32*), and its product itaconate inhibits mycobacterial growth (*33*) and contributes to macrophage control of the intracellular pathogens *Salmonella enterica* (*33*) and *L. pneumophila* (*34*). However, a role in host defense against extracellular gram-positive bacteria has not been reported to date. First, we demonstrated that macrophage *ACOD1* expression is induced in response to several gram-positive species (Fig. 3B) and that itaconate was directly microbicidal against pneumococci at the physiologically relevant concentration of 5 mM (Fig. 3C) (*33*). This effect was partially pH dependent, but pH-neutralized itaconate and the pH neutral ester dimethyl itaconate retained microbicidal activity against pneumococci (fig. S4A). The microbicidal effect of itaconate extended to *Streptococcus agalactiae*, *Streptococcus pyogenes*, and vancomycin-resistant *Enterococcus faecium* ST262 (VREfm), whereas *S. aureus* was relatively resistant (Fig. 3C). Administration of exogenous itaconate at a concentration known to penetrate macrophages [10 mM; (*35*)] increased hMDM early intracellular killing of pneumococci after *ACOD1* siRNA knockdown (Fig. 3D) and also increased killing of *S. agalactiae*, *S. pyogenes*, and VREfm ST262, but not *S. aureus* (Fig. 3E). pH neutral dimethyl itaconate also increased hMDM intracellular bacterial killing (fig. S4B). To validate these findings in an in vivo system, we first generated murine bone marrow–derived macrophages (mBMDMs) from *Acod1*^−/−^ mice. Consistent with our hMDM findings, *Acod1^−/−^* BMDM demonstrated normal phagocytosis (Fig. 3F) but impaired early killing of pneumococci (and unaltered killing of *S. aureus*; Fig. 3G). Furthermore, pneumococcal killing by mBMDM was rescued by supplementation with exogenous itaconate (Fig. 3H). After intraperitoneal bacterial challenge, *Acod1*^−/−^ mice were more susceptible to bacteraemia and recruited more neutrophils to the peritoneal cavity to effect bacterial clearance (Fig. 3, I and J). Our results therefore extend the role of ACOD1 and its product itaconate to defense against extracellular gram-positive bacteria, in addition to pathogens with a predominantly intracellular lifestyle.

### NAMPT mediates host defense against pneumococci

**Fig. 4.**
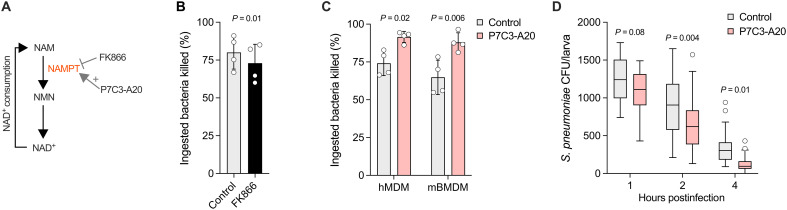
NAMPT activity supports killing of *S. pneumoniae* by macrophages and zebrafish. (**A**) Schematic representation of NAD^+^ salvage involving NAMPT. NAM, nicotinamide; NMN, nicotinamide mononucleotide; NAD^+^, nicotinamide adenine dinucleotide (oxidized form); NAMPT, nicotinamide phosphoribosyltransferase. (**B**) Effect of 100 nM FK866 treatment on hMDM early intracellular killing of *S. pneumoniae* ST618 MOI of 10 (*n* = 4 biological replicates), compared using paired *t* test. (**C**) Effect of 5 μM P7C3-A20 treatment on hMDM and mBMDM early intracellular killing of *S. pneumoniae* ST618 MOI of 10 (*n* = 4 biological replicates), compared using paired *t* tests. (**D**) Effect of 10 μM P7C3-A20 treatment on zebrafish larva clearance of *S. pneumoniae* D39 Δ*cps* after intravenous microinjection of 1600 CFU (*n* = 28 to 32 larvae per group). Data presented as Tukey box-and-whisker plot, compared using ANOVA with Šidák’s multiple comparisons test. [(B) and (C)] Data presented as means and SD; data points represent biological replicates.

NAMPT was ranked 17 of 828 genes involved in the host response to pneumococci prioritized by MAIC. NAMPT is required for nicotinamide adenine dinucleotide (oxidized form) (NAD^+^) salvage in inflammatory macrophages (Fig. 4A) (*36*) but has no known role in host antibacterial defense. To address this, we inhibited NAMPT in hMDMs using the specific inhibitor FK866, demonstrating reduced early bacterial killing (Fig. 4B). Furthermore, treatment of both hMDMs and mBMDMs with the NAMPT activator P7C3-A20 (*37*) increased early killing of pneumococci (Fig. 4C). We confirmed this in vivo (Fig. 4D), using an established zebrafish larval model of pneumococcal infection that involves macrophage clearance of bacteria after intravenous microinjection (*20*). NAMPT therefore represents a druggable mediator of host antibacterial defense identified by our approach.

### Clemastine is a macrophage-based P2RX7-dependent host-directed therapy

**Fig. 5.**
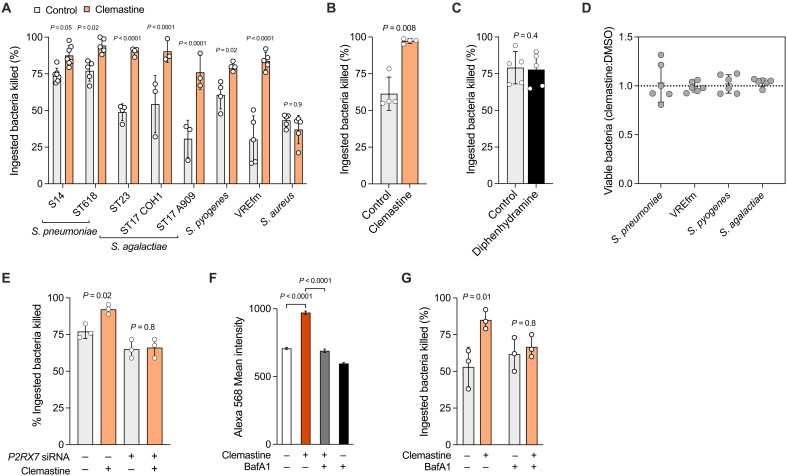
Clemastine augments phagolysosomal killing of gram-positive bacteria by macrophages. (**A**) Effect of 10 μM clemastine treatment on hMDM early intracellular killing of gram-positive bacteria (*n* = 3 to 7 biological replicates), compared using ANOVA with Šidák’s multiple comparisons test. Pneumococci were used at MOI of 10, and all other bacteria were used at MOI of 5. The *S. aureus* strain used was Newman. (**B**) Effect of 10 μM clemastine treatment on mBMDM early intracellular killing of *S. pneumoniae* ST618 MOI of 10 (*n* = 4 biological replicates), compared by paired *t* test. (**C**) Effect of the H_1_-antihistamine diphenhydramine (10 μM) on hMDM early intracellular killing of *S. pneumoniae* ST618 MOI of 10 (*n* = 4 biological replicates), compared by paired *t* test. (**D**) Effect of 10 μM clemastine on viability of planktonic bacteria (*n* = 6 technical replicates). Bacterial viability after 1 hour, compared to DMSO, is shown as a ratio. *S. pneumoniae* strain used was ST618, and *S. agalactiae* was ST23. (**E**) Effect of *P2RX7* siRNA knockdown and 10 μM clemastine on hMDM early intracellular killing of *S. pneumoniae* ST618 MOI of 10 (*n* = 3 biological replicates), compared using ANOVA with Šidák’s multiple comparisons test. (**F**) DQ-Red BSA staining intensity in bacteria-containing hMDM (*n* = 3 biological replicates, mean 684 cells counted per donor), compared using Kruskal-Wallis test with Dunn’s multiple comparisons test. (**G**) Effect of 10 μM clemastine and 100 nM bafilomycin A1 on hMDM early intracellular killing of *S. pneumoniae* ST618 MOI of 10 (*n* = 3 biological replicates), compared using ANOVA with Šidák’s multiple comparisons test. [(A) to (G)] Data presented as means and SD; data points represent biological replicates.

**Fig. 6.**
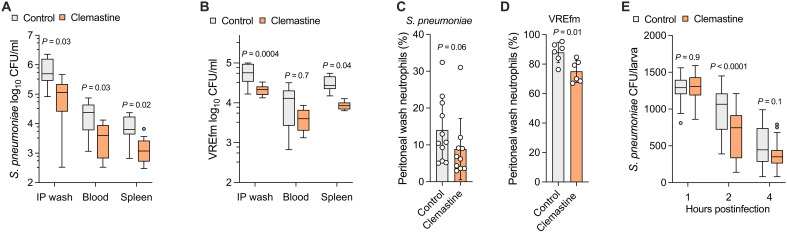
Clemastine increases bacterial clearance by mice and zebrafish. Effect of clemastine treatment (10 mg/kg) on wild-type mouse clearance of (**A**) 10^3^ CFU *S. pneumoniae* ST227 INV104B (*n* = 10 to 11 female mice per group) and (**B**) 10^9^ CFU VREfm ST262 (*n* = 6 female mice per group) after intraperitoneal infection, compared using ANOVA with Šidák’s multiple comparisons test. Percentages of peritoneal wash neutrophils in the mice infected with (**C**) *S. pneumoniae* and (**D**) VREfm ST262, compared using unpaired *t* test. (**E**) Effect of 10 μM clemastine treatment on zebrafish larva clearance of *S. pneumoniae* D39 Δ*cps* after intravenous microinjection of 1600 CFU (*n* = 26 to 28 larvae per group), compared using ANOVA with Šidák’s multiple comparisons test. [(A), (B), and (E)] Data presented as Tukey box-and-whisker plot. [(C) and (D)] Data presented as means and SD; data points represent biological replicates.

The P2RX7 senses extracellular adenosine 5′-triphosphate (ATP) and other nucleotides to activate immune cells, especially macrophages (*38*). Pharmacological modulation of signaling via P2RX7 altered hMDM early pneumococcal killing (enhanced by the agonist bz-ATP; inhibited by the antagonist KN-62; fig. S5A). Clemastine, a licensed H_1_-antihistamine drug with off-target P2RX7 potentiation (*19*), enhanced hMDM and mBMDM early killing of pneumococci (Fig. 5, A and B). In hMDMs, we confirmed this effect extended to *S. agalactiae*, *S. pyogenes*, and VREfm ST262, but not to *S. aureus* (Fig. 5A). The effect was specific to clemastine (not observed with another H_1_-antihistamine, diphenhydramine; Fig. 5C) was not due to direct antimicrobial activity (Fig. 5D) and was *P2RX7* dependent (Fig. 5E). Network analysis indicated *P2RX7* was coexpressed with several genes involved in phagolysosome maturation, including *LAMP3* (fig. S5B). Mechanistically, clemastine increased phagolysosomal pH-dependent protease degradative capacity (Fig. 5F). Inhibition of phagolysosome acidification with the vacuolar adenosine triphosphatase inhibitor bafilomycin A1 (*20*) abrogated the effect of clemastine on early killing (Fig. 5G). In murine models of *S. pneumoniae* ST227 INV104B (Fig. 6A) and VREfm ST262 (Fig. 6B) disease established by intraperitoneal injection, clemastine pretreatment enhanced bacterial clearance in the peritoneal cavity and during bacteraemia and reduced the requirement for neutrophil recruitment to the peritoneal cavity (Fig. 6, C and D). The higher proportion of neutrophils observed in the VREfm-challenged mice likely reflects the higher bacterial dose required to establish infection (Fig. 6, C and D). Clemastine also enhanced clearance of pneumococci by zebrafish larvae after intravenous microinjection (Fig. 6E). Overall, this provides proof of concept that a pathogen-centric approach can identify therapeutically targetable mediators of host defense against gram-positive bacteria and can be used as a strategy to repurpose drugs to target these responses.

## DISCUSSION

In this study, we exploited naturally occurring variation within serotype 1 pneumococci, associated with differential susceptibility to macrophage intracellular killing, to identify host microbicidal factors contributing to pathogen clearance. Distinct transcriptional programs were identified in association with differential killing of the serotype 1 pneumococcal variants. In addition to canonical mediators of host defense (e.g., *IFNG*), it was notable that enriched pathways and differentially expressed genes often had known roles in defense against classic intracellular/intramacrophagic pathogens such as mycobacteria [*ACOD1* (*33*), *P2RX7* (*39*), *SPHK1* (*40*), *NPC2* (*41*), *HCAR2* (*42*), *IL23* (*43*), *IL27* (*44*), and *XCL1* (*45*)], *Burkholderia pseudomallei* [*SPHK1* (*46*)], *S. enterica* [*ACOD1* (*33*)], *L. pneumophila* [*ACOD1* (*34*) and *IL12* (*47*)], and *Listeria monocytogenes* [*GZMB* (*48*)], whereas pneumococci are traditionally considered to be extracellular pathogens. This further supports the revised paradigm that the intracellular phase of pneumococcal infection is a key determinant of the outcome of infection (*16*–*18*) and that the dichotomization of intra- versus extracellular risks neglecting critical host responses. Host responses canonically associated with control of intracellular pathogens such as mycobacteria are very likely to have relevance to a broader range of bacterial pathogens. Although our findings extended from pneumococci to related streptococci and enterococci, *S. aureus* was not susceptible to these responses. This is consistent with the increasingly recognized adaptation of *S. aureus* to the intracellular niche and phagolysosome specifically (*49*).

Our results reveal three microbicidal responses active against a range of medically important gram-positive bacteria. ACOD1-mediated itaconate production is likely to be a direct microbicidal mechanism in macrophages in response to streptococci and enterococci. Itaconate inhibits isocitrate lyase in mycobacteria to inhibit the glyoxalate shunt (*33*), but this is not a metabolic process used by streptococci (*50*). Furthermore, itaconate was found to be directly bactericidal, not bacteriostatic, consistent with a direct toxic effect. We identify NAMPT as a microbicidal factor and demonstrate that bacterial clearance could be enhanced in vivo with the NAMPT activator P7C3-A20, representing an investigational target for host-directed therapy. It is plausible that NAMPT enhances glycolytic metabolism to support intracellular bacterial killing, and this process is augmented by P7C3-A20. Macrophages perform glycolysis to activate effector mechanisms in response to bacteria (*51*). This transition to glycolytic metabolism has been demonstrated specifically for pneumococci (*52*), requiring NAD^+^, which, in turn, requires NAMPT for NAD^+^ salvage (*36*). In murine alveolar macrophages, enhancing glycolysis via addition of exogenous acetate is associated with increased killing of pneumococci (*53*). P2RX7 signaling was identified as a regulator of the macrophage microbicidal response against pneumococci. P2RX7 could be targeted using clemastine, representing a candidate for repurposing as a host-directed therapy against streptococci and enterococci, subject to confirmation that this host-directed microbicidal effect occurs during human infection. Antimicrobial options for VREfm infections are extremely limited, so the finding that clemastine-increased bacterial clearance in vivo offers a timely approach to an unmet clinical need, warranting further investigation. As evidenced by reduced neutrophil recruitment to the site of infection, augmented killing did not occur at the expense of excessive inflammation, suggesting attainment of “pauci-inflammatory” killing with less capacity for secondary tissue damage (*54*).

Together, our study demonstrates that pathogen-centric host screening, using carefully selected immune-adaptive pathogen variants, is an efficient and powerful method for identifying critical host defense mechanisms. In addition to serotype 1 pneumococci, other examples of such variants include the *S. aureus* USA300 clone associated with metastatic complications during bacteraemia (*55*), the recently emerged toxigenic M1_UK_ *S. pyogenes* lineage (*56*), an *Enterobacter cloacae* complex species associated with neonatal septic shock (*57*), hypermucoviscous *Klebsiella pneumoniae* associated with liver abscesses and metastatic complications (*58*), and the rapidly adaptable W-Beijing lineage of *M. tuberculosis* (*59*). This study focused on the differences in host responses elicited by immune-adaptive serotype 1 isolates, but not the pathogen characteristics underlying these differences. A bacterial genome wide association study, using escape from macrophage killing as the trait, would be an attractive methodology to address this question but would require a much larger sample size of isolates and a more high-throughput assay to assess macrophage intracellular killing.

Determining the significance of our findings in humans with gram-positive bacterial infections remains a major bottleneck in the pathway to establishing clinical relevance. First, we used hMDMs and mBMDMs in our macrophage experiments but recognize that these are ontogenically distinct from populations of tissue macrophages likely to be important during human infection (*60*), for example, alveolar, splenic, and hepatic macrophages. However, hMDMs, in particular, are an accepted model of tissue macrophages and exhibit similar phenotypic properties, for example, compared to alveolar macrophages in COPD (*61*). Second, the gap between potential host-directed therapies with experimental evidence versus translational efficacy remains large (*54*). However, proof of principle of the approach does exist, for example, prevention of serious infection in people with chronic granulomatous disease using IFN-γ (*62*). We contend that this large translational gap supports the importance of diverse discovery approaches such as the pathogen-centric approach described here.

We validated *NAMPT*, *ACOD1*, and *P2RX7* as genes regulating macrophage microbicidal responses, identified through pathogen-centric screening using hypervirulent serotype 1 pneumococci. These represent targets for host-directed therapies, including drug repurposing. Consistent with this, we identified the H_1_-antihistamine clemastine as a candidate for drug repurposing to augment macrophage phagolysosomal bacterial killing in streptococcal and VREfm infections. Overall, we demonstrate the traction of a pathogen-centric approach to identify macrophage microbicidal responses against bacteria and associated candidates for host directed therapeutic targeting. This provides a basis for translational medicine studies, including through the use of repurposed drugs.

## MATERIALS AND METHODS

### Study design

The objective of this study was to identify mediators promoting bacterial killing by macrophages and identify targets for host-directed therapies. To do this, we studied the interaction between hypervirulent serotype 1 pneumococci and macrophages to identify microbicidal responses a successful pathogen variant has evolved to escape. Eleven clinical isolates of serotype 1 pneumococci recovered from invasive disease were obtained and underwent whole-genome sequencing. Primary hMDMs obtained from healthy donors were challenged with bacteria, and intracellular bacterial killing was quantified using a modified gentamicin protection assay. hMDM transcriptional responses were analyzed by bulk RNA-seq. siRNA transfection was used for gene knockdown in hMDMs. Pharmacologic manipulation of pathways of interest was performed in hMDMs and mBMDMs. For in vitro macrophage experiments, two technical replicates were performed for each condition and at least three biological replicates (i.e., cells from different donors). For in vivo validation, intraperitoneal injection of bacteria into C57BL/6J mice was used to establish systemic infection, using both wild-type and *Acod1*^−/−^ mice. Zebrafish larvae were infected with unencapsulated *S. pneumoniae* at 36 hours postfertilization by microinjection to establish a macrophage-controlled model of pneumococcal infection. In experiments involving drug treatments, allocation to treatment or control was random. Sample sizes were determined on the basis of previous similar experiments and are provided in the figure legends. No data points were excluded.

### Bacteria and growth conditions

Bacterial isolates used in this work are detailed in tables S2 and S3. Bacterial stocks were grown in brain-heart infusion broth (Oxoid) supplemented with 20% sterile-filtered, heat-inactivated low-lipopolysaccharide (LPS) fetal bovine serum (FBS; PAN-Biotech). For pneumococci, *S. aureus* Newman and USA300, and *S. agalactiae*, stocks were prepared at midlog phase [optical density at 600 nm (OD_600_) ~ 0.5] and for *S. aureus* SH1000 at stationary phase (OD_600_ ~ 1.0). For *S. pyogenes*, a single colony from overnight growth on blood agar was inoculated into brain-heart infusion broth and 20% FBS and grown overnight at 37°C without shaking. The broth was then subcultured 1:10 the next morning (into brain-heart infusion broth and 20% FBS) and grown without shaking until midlog phase. Stocks were stored at −80°C before use in experiments. For some experiments, bacteria were heat killed by incubation in water at 60°C for 40 min. For experiments assessing the viability of planktonic bacteria, 10^5^ colony-forming units (CFU) of bacteria were incubated in phosphate-buffered saline (PBS) with/without the compound of interest for the specified time period, and, then, the remaining viable bacteria were quantified by surface viable counts (*63*) on Columbia blood agar (Oxoid) containing 5% defibrinated horse blood (E&O Laboratories Limited).

### Bacterial whole-genome sequencing and phylogenetic analysis

DNA extraction, library preparation, and sequencing were performed by MicrobesNG, University of Birmingham [Illumina HiSeq 2500 platform with a 250–base pair paired-end protocol followed by adapter trimming using Trimmomatic 0.30 and then de novo assembly using SPAdes version 3.7; (*64*, *65*)]. Sequenced *S. pneumoniae* isolates with complete genome assemblies available were identified and downloaded from National Center for Biotechnology Information (*n* = 128), including a serotype 1 isolate with a completed annotated genome (GCA_001457635.1 NCTC7465), which was used as the reference genome. Parsnp (*66*) was used to produce a preliminary core genome single-nucleotide polymorphism (SNP) tree including the sequenced isolates from this study (as FASTA format SPAdes assemblies) and this collection of available pneumococcal sequences. Protein-coding sequences within the study isolate assemblies were then annotated using Prokka (*67*). Snippy was used to align and call SNPs in the assemblies against the serotype 1 reference genome. Snippy-core was then used to discard non–core genetic areas and create a core genome SNP alignment. Gubbins (*68*) was used to identify loci of recombination in the core genome SNP alignment, which were removed using Maskrc and SNP sites (*69*). The resulting core genome SNP alignment, stripped of recombinant regions, was used as input for FastTree (*70*) to infer a maximum-likelihood phylogenetic tree which was visualized using Figtree.

### Isolation and culture of macrophages

Primary human peripheral blood mononuclear cells (PBMCs) were isolated from whole blood donated by healthy male and female volunteers. PBMCs were isolated by dextran sedimentation followed by discontinuous Percoll gradient centrifugation, as previously described (*71*). PBMCs (2.5 × 10^6^) were added to wells in a 24-well tissue culture treated flat-bottomed polystyrene plate (Corning Costar) in RPMI 1640 (Sigma-Aldrich) supplemented with 10% autologous serum and l-glutamine (2 mM). Nonadherent cells were removed after 3 hours, and the culture medium was replaced with RPMI 1640 supplemented with l-glutamine and 10% low-LPS FBS. Adherent cells were cultured for ≥14 days to differentiate into monocyte-derived macrophages (hMDMs; final concentration, 2 × 10^5^ per well).

Murine bone marrow was obtained, and cells were cultured in Dulbecco’s modified Eagle’s medium containing d-glucose (4.5 g/liter) and sodium pyruvate (0.11 g/liter), supplemented with 10% FBS, 1:100 100× GlutaMAX (l-alanyl-l-glutamine dipeptide; Gibco), penicillin (100 U/ml), streptomycin (0.1 mg/ml), and CSF-1 (100 ng/ml) in 100-mm square Sterilin plastic plates at 37°C for ≥7 days as previously described (*12*). Before use, bone marrow–derived macrophages (mBMDMs) were resuspended in medium without antimicrobials and cultured overnight in 24-well plates at a final concentration of 5 × 10^5^ mBMDMs per well.

### Intracellular bacterial killing assay

Bacteria were opsonized with either 10% human immune serum from a recipient of the protein conjugate pneumococcal vaccine (hMDM) or 10% murine immune serum from immunized mice with detectable antipneumococcal antibodies (mBMDM) (*21*). After addition of bacteria (multiplicity of infection used for each experiment is stated in the figure legends), macrophages were incubated for 1 hour on ice and then 3 hours at 37°C as previously described (*72*). The remaining extracellular bacteria were removed with PBS washes and a 30-min incubation with either benzyl-penicillin (40 IU/ml; Sigma-Aldrich) and gentamicin (20 μg/ml; Lonza) for streptococci and *E. faecium* or vancomycin (0.75 μg/ml; Sigma-Aldrich) and gentamicin for *S. aureus*. At the required time points, cells were lysed with 2% saponin (in PBS) and viable intracellular bacteria quantified by surface viable counts (*63*) on Columbia blood agar (Oxoid) containing 5% defibrinated horse blood (E&O Laboratories Limited). Cells were incubated with vancomycin until subsequent time points. In all experiments, viable intracellular bacteria were quantified after removal of extracellular bacteria after the 4-hour challenge period to determine the initial ingested load. In experiments assessing early intracellular killing, viable intracellular bacteria were quantified again after a further 1 hour, and the percentage of bacteria killed was calculated relative to the initial ingested load.

YVAD-FMK (10 μM; Calbiochem), recombinant human IL-1RA (200 ng/ml; PeproTech), or KN-62 (1 μM; Sigma-Aldrich) was added 1 hour before bacterial challenge and then maintained in the medium. P7C3-A20 (5 μM; Cayman Chemical), FK866 (100 nM; APExBIO), itaconate (10 mM; Merck), citrate (10 mM; Fisher Scientific), dimethyl itaconate (10 mM; Sigma-Aldrich), bz-ATP (3 mM; Sigma-Aldrich), clemastine (10 μM; Sigma-Aldrich), or diphenhydramine (10 μM; Sigma-Aldrich) was added after removal of extracellular bacteria and then maintained in the medium. Human recombinant IFN-γ (50 ng/ml; STEMCELL Technologies Inc.) was added overnight before bacterial challenge and then not maintained in the medium. Bafilomycin A1 (100 nM; Stratech Scientific Ltd.) was added 2 hours before bacterial challenge and then not maintained in the medium.

### hMDM transcriptional profiling

Four hours after bacterial challenge, hMDM RNA was extracted using the QIAGEN RNeasy Plus kit. RNA was quantified using a NanoDrop spectrophotometer, and integrity was assessed using a LabChip GX Touch 24 Nucleic Acid Analyzer (mean quality score, 9.5; SD, ±0.3). Illumina TruSeq stranded mRNA-seq libraries were prepared and then sequenced using the Illumina NovaSeq 6000 platform by Edinburgh Genomics. Salmon was used to map trimmed unaligned reads to a reference transcriptome and quantify transcript abundance (*73*). Differential expression analysis was performed using the edgeR package in RStudio (*74*). The experiment had a paired design: MDMs from each independent donor were challenged with both bacterial isolates. Therefore, an additive model without an interaction term was specified. Preranked gene set enrichment analysis of differentially expressed genes was performed using the Fast Gene Set Enrichment Analysis (FGSEA) package in RStudio (*75*).

### Meta-analysis by information content

The MAIC algorithm aggregates gene lists from genome-scale studies to generate a single ranked output list, where a gene’s ranking reflects the cumulative evidence supporting the gene (*31*, *76*). The algorithm has been previously shown to outperform other ranking aggregation methods (*30*). Source code and documentation are available at https://github.com/baillielab/maic. The unit invariant knee method was used to determine the “elbow point” in the distribution of MAIC scores to identify genes prioritized by the analysis. Details of input lists are shown in table S4.

### siRNA knockdown

Predesigned mixtures of four siRNA per target gene, or a nontargeting control pool, were used for each transfection (ON-TARGETplus SMARTPools, Horizon Discovery Limited). siRNA lipid complexes were prepared by incubating siRNA with Lipofectamine RNAiMAX transfection reagent (Invitrogen) in Opti-MEM reduced-serum medium (Gibco). hMDMs were then transfected with 100 nM of pooled target or pooled control siRNA and incubated for 4 hours before the medium was replaced with RPMI 1640 with 10% FBS to allow the cells to recover for 16 to 20 hours. This was performed a total of three times on consecutive days before use in bacterial killing assays. Reduction of target gene transcript abundance relative to *GAPDH* was confirmed by reverse transcription quantitative polymerase chain reaction (fig. S6).

### Assessment of phagolysosomal degradative capacity

hMDMs (≥14 days old) were replated at 150,000 cells per well into eight-well chamber slides (ibidi). Cells were incubated with DQ-Red BSA (10 μg/ml) (*77*) with/without 100 nM bafilomycin A1 (to prevent phagolysosomal acidification) for 12 hours before bacterial challenge. After 1 hour on ice and then 3 hours at 37°C, extracellular bacteria were removed, and 10 μM clemastine or dimethyl sulfoxide (DMSO) control was added to the medium. After 1.5 hours, cells were fixed with paraformaldehyde (2%), stained with 4′,6-diamidino-2-phenylindole (DAPI; 1 μg/ml), and then imaged on the Opera Phenix Plus system (Revvity). *Z*-stacks (1-μm stacks, ×20 planes, ×20 fields) were obtained in confocal mode using a 40× water objective lens (numerical aperture, 1.1). For DAPI, exposure was 200 ms, power was 70%, excitation laser was 375 nm, and emission filter was 435 to 480 nm. For Alexa Fluor 568 (DQ-Red BSA), exposure was 400 ms, power was 90%, excitation laser was 561 nm, and emission filter was 570 to 630 nm. Images were analyzed using Harmony 5.2 software (Revvity). Unquenched DQ-Red BSA fluorescence was quantified per bacteria-containing whole cell, defined using DAPI to identify nuclei and intracellular bacteria.

### Mouse infection model

Eight- to 12-week-old wild-type C57BL/6 (Charles River Laboratories) or C57BL/6NJ-*Acod1*^em1(IMPC)3^ (referred to as *ACOD1^−/−^*) mice were used in this study. *ACOD1^−/−^* mice were provided by A. Byrne (Imperial College, London) who originally obtained them from the Jackson Laboratory. Mice were housed in specific pathogen–free conditions before infection. Infection was established by intraperitoneal instillation of 100 μl of PBS containing bacteria opsonized with murine immune serum (bacterial doses stated in the figure legends). After 24 hours (*S. pneumoniae* ST227 INV104B) or 14 hours (VREfm ST262), mice were anesthetized and then killed for collection of peritoneal wash fluid, spleen, and blood (obtained by cardiac puncture). The spleen was homogenized [using CK14 ceramic beads (VWR) in a Precellys tissue homogenizer], and, then, viable bacteria in all compartments were quantified by plating on Columbia blood agar (Oxoid) containing 5% defibrinated horse blood (E&O Laboratories Limited). The proportion of neutrophils in the peritoneal wash fluid was determined by analysis of Cytospin preparations after Kwik-Diff staining (Thermo Shandon), as previously described (*12*). For experiments involving clemastine treatment, mice received an intraperitoneal injection of either clemastine (10 mg/kg) or DMSO vehicle control once daily on days 1 to 7 (*78*, *79*), followed by bacterial infection on day 8.

### Zebrafish larvae infection model

Wild-type ABTL zebrafish larvae were incubated in E3 medium at 28°C in accordance with the standard protocols (*80*). At 34 hours postfertilization, larvae were bath treated with either 10 μM clemastine (Sigma-Aldrich), 10 μM P7C3-A20 (Cayman Chemical), or 0.1% DMSO as vehicle control (Sigma-Aldrich) in E3 medium for 2 hours before infection. At 36 hours postfertilization, ~1600 CFU of *S. pneumoniae* D39 Δ*cps* were microinjected intravenously into larvae. Following microinjection, larvae were transferred to E3 medium containing clemastine, P7C3-A20, or DMSO. At 1, 2, and 4 hours postinfection, ~10 anesthetized zebrafish larvae from treated and control groups were individually transferred into 0.5-ml tubes and then homogenized. The homogenates were plated onto tryptic soy agar containing 5% defibrinated sheep blood to quantify viable bacteria. The limit of detection was 10 CFU per larva. As previously described, this approach establishes a macrophage controlled model of systemic pneumococcal infection (*20*). The Δ*cps* mutant was used, first, to maximize macrophage phagocytosis and intracellular bacterial numbers, stressing intracellular bacterial killing to test the impact of compounds and, second, to avoid causing rapidly overwhelming infection.

### Ethics statement

Blood donations from healthy volunteers were obtained through the University of Edinburgh Centre for Inflammation Research blood resource, with ethical approval granted by the Edinburgh Accredited Medical Regional Ethics Committee (21-EMREC-041). Volunteers provided written informed consent. Mouse experiments were performed under project license PPL PP8738752 in accordance with UK Home Office regulations [Animals (Scientific Procedures) Act 1986]. Ethical approval was granted by the University of Edinburgh’s Protocols and Ethics Committee. The animal care and use protocols adhered to the National Centre for the Replacement, Refinement and Reduction of Animals in Research guidelines [Responsibility in the use of animals in bioscience research, April 2019, and Animal Research: Reporting of In Vivo Experiments (ARRIVE) guidelines, June 2010]. Zebrafish experiments were conducted in accordance with the European Community Council Directive 2010/63/EU for the Care and Use of Laboratory Animals of 22 September 2010 (Chapter 1, Article 1 no. 3) and National Journal of Law act of 15 January 2015 for Protection of animals used for scientific or educational purposes (Chapter 1, Article 2 no. 1). All experiments with zebrafish were performed on embryos/larvae up to 48 hours postfertilization and followed ARRIVE guidelines. The Jagiellonian University Zebrafish Core Facility is a licensed breeding and research facility (District Veterinary Inspectorate in Krakow registry; Ministry of Science and Higher Education record nos. 022 and 0057). Collection of the *E. faecium* isolate VRE-EDI-160 was approved by the NHS Scotland Biorepository Network (TR000126).

### Statistical analysis

Before statistical comparisons, data distributions were assessed by the Shapiro-Wilk test. Groups of normally distributed data were compared by *t* test or analysis of variance (ANOVA) as appropriate, or, if not normally distributed, Mann-Whitney or Kruskal-Wallis tests were used. When multiple comparisons were made, this was controlled by use of multiple comparison tests and adjusted *P* values reported. When macrophages from the same donor or mouse were exposed to different conditions, a paired test was used to compare the conditions. All reported *P* values are two-sided. Specific tests and sample sizes are detailed in the figure legends. Analyses and data visualization were done using GraphPad Prism version 10.4.1 for macOS.

## Supplementary Materials

### The PDF file includes:

Figs. S1 to S6 Tables S1 to S4 Legends for data files S1 to S3 References
