# Supplementary material for: Immune-adaptive pathogen variation reveals targetable mediators of gram-positive bacterial killing in macrophages
Source: Sci Adv. 2026 Feb 27;12(9):eaea0375. doi: 10.1126/sciadv.aea0375 (PMC12947864; doi:10.1126/sciadv.aea0375)
Supplement: Supplementary file 1 — Figs. S1 to S6 Tables S1 to S4 Legends for data files S1 to S3 References [file sciadv.aea0375_sm.pdf]

Science Advances
AAAS

Supplementary Materials for

Immune-adaptive pathogen variation reveals targetable mediators of gram-positive bacterial killing in macrophages

Clark D. Russell *et al.*

Corresponding author: David H. Dockrell, david.dockrell@ed.ac.uk

Sci. Adv. **12**, eaea0375 (2026)
DOI: 10.1126/sciadv.aea0375

The PDF file includes:

Figs. S1 to S6
Tables S1 to S4
Legends for data files S1 to S3
References

Other Supplementary Material for this manuscript includes the following:

Data files S1 to S3

SUPPLEMENTARY MATERIALS

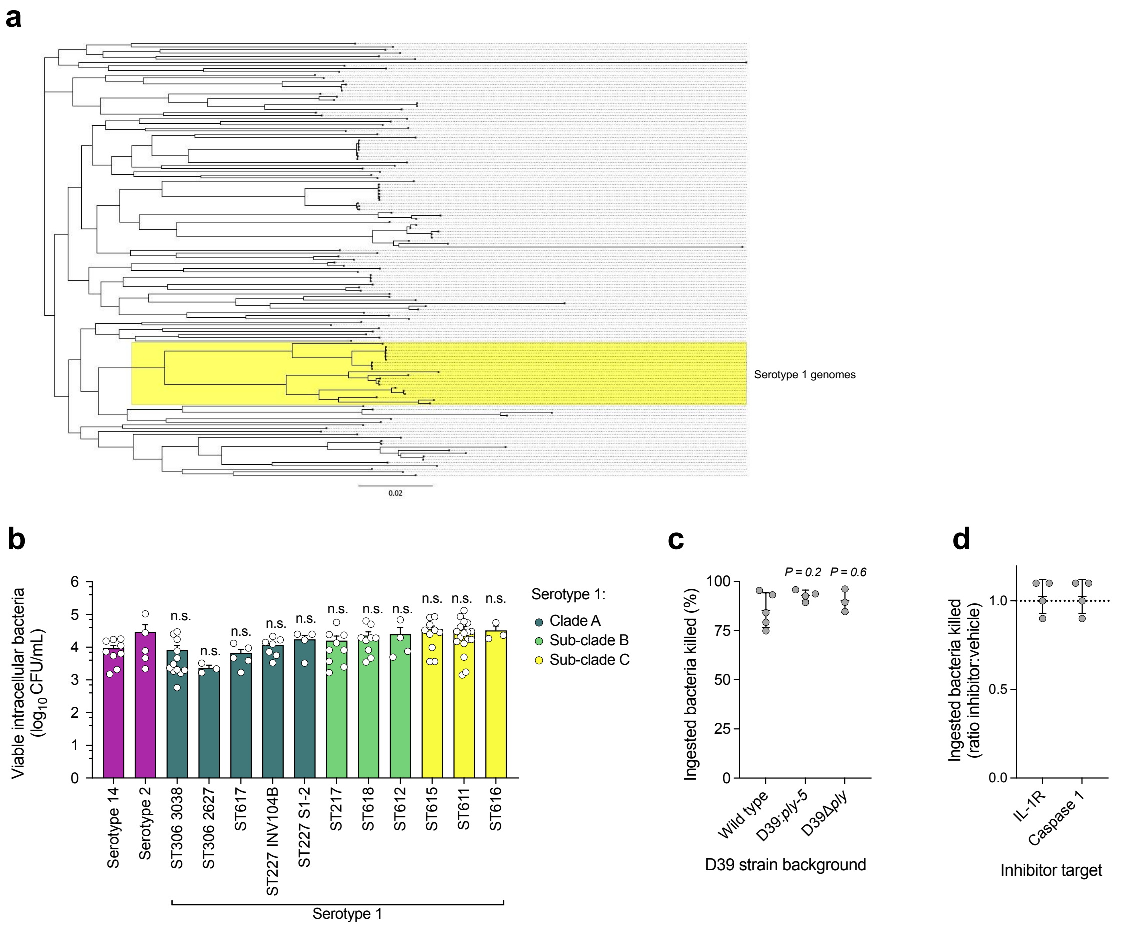

Supplementary Fig. 1: Screening *S. pneumoniae* serotype 1 isolates for susceptibility to macrophage intracellular killing.

(A) Core genome SNP tree produced using Parsnp and visualised with Figtree illustrating the relationship of the serotype 1 study isolates with the pneumococcal population using 128 complete genome assemblies downloaded from NCBI. All study isolates and 10 reference genomes from serotype 1 pneumococci clustered together in the clade highlighted in yellow. Scale bar indicates number of nucleotide changes per site. **(B)** Viable intracellular bacteria after 4h challenge period (n=3-17 biological replicates). Mean intracellular viable counts of serotype 1 isolates were compared to serotype 14 using ANOVA with Dunnett's multiple comparisons test. n.s. not significant ($P > 0.05$). A multiplicity of infection (MOI) of 20 was used for ST306 3038, ST306 2627 and ST617; MOI=5 for ST615, ST611 and ST616; and MOI=10 for all other isolates. Data presented as mean and standard deviation. **(C)** Effect of pneumolysin variation on hMDM early intracellular bacterial killing, MOI=10 (n=3-4 biological replicates). Mutants compared to wild type *S. pneumoniae* D39 using ANOVA with Dunnett's multiple comparisons test. **(D)** Effect of pre-treatment with recombinant human IL-1RA or YVAD, to inhibit IL-1R or caspase 1 respectively, on hMDM early intracellular killing of *S. pneumoniae* D39 MOI=10 (n=4 biological replicates). Graph shows ratio of ingested bacteria killed pre-treated with inhibitor relative to vehicle. A ratio of 1.0 (dotted line) indicates no difference between inhibitor and vehicle. (C-E) Data presented as mean and standard deviation.

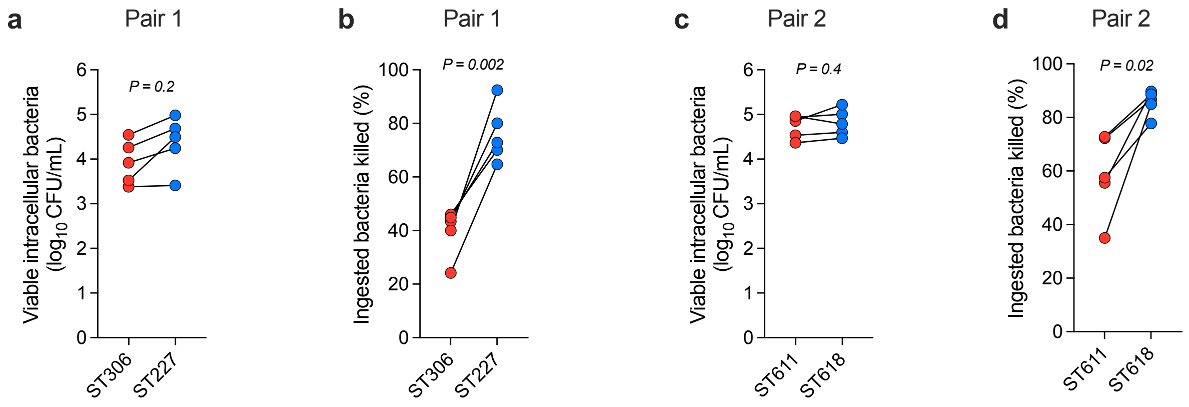

Supplementary Fig. 2: Bacterial phagocytosis and killing in experiments used to generate RNA for RNAseq. Viable intracellular bacteria after 4h challenge (**A,C**) and hMDM early intracellular bacterial killing (**B,D**) in experiments used to generate RNA. Data points represent biological replicates (n=5), compared by paired t-test. Lines connect data points from same donor.

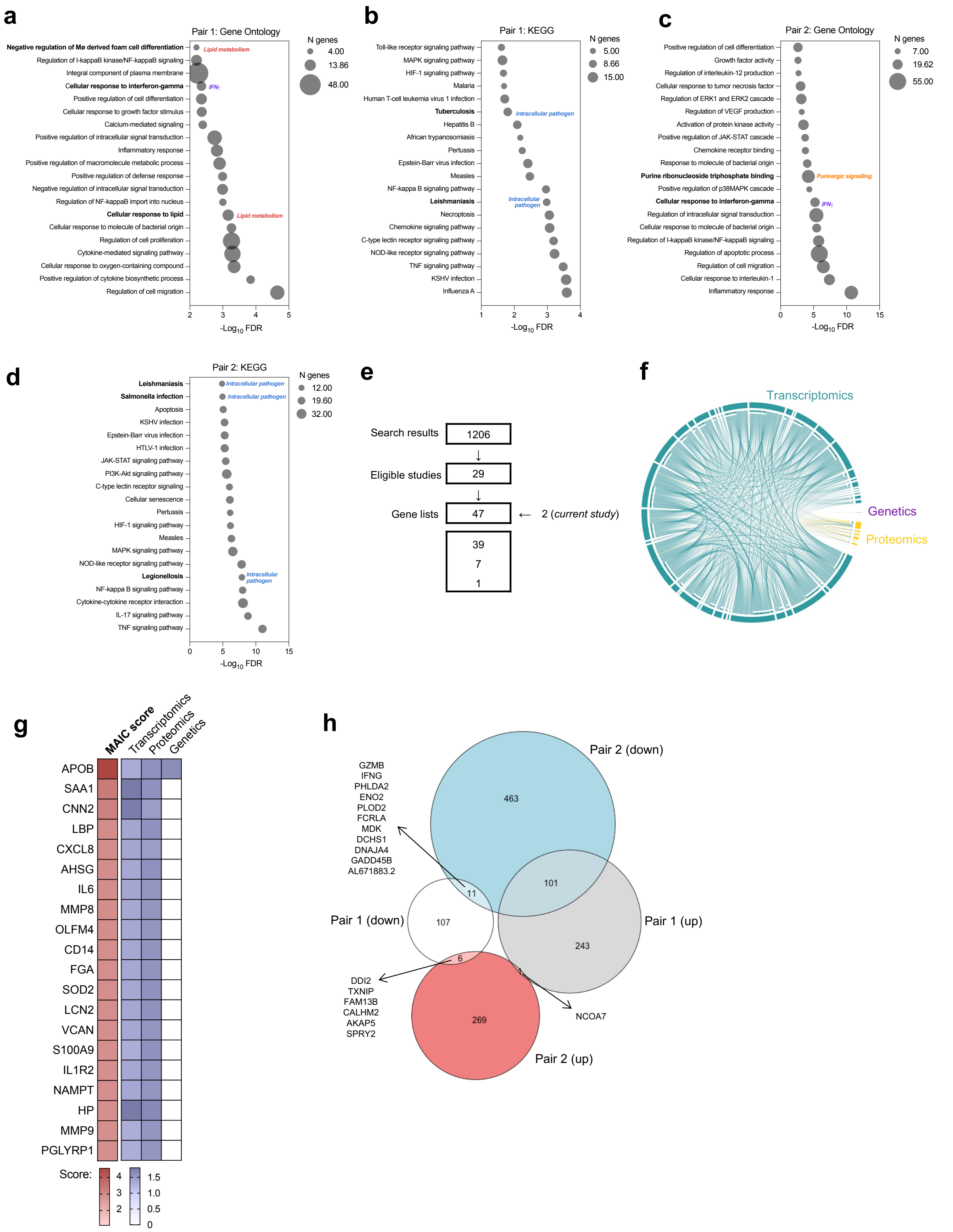

Supplementary Fig. 3: Prioritisation of differentially expressed macrophage genes.
(A-D) Gene set enrichment of differentially expressed genes (false discovery rate <0.05). **(E)** Identification of input data for MAIC. **(F)** Shared information between gene lists and weighting of experimental categories. The links in the plot indicate the sum of common gene scores between lists. **(G)** Heatmap showing the experimental categories contributing to the overall MAIC scores of the 20 highest ranked genes. Intensity of shading correlates with strength of evidence (MAIC score). **(H)** Overlapping differentially expressed genes between pairs. Pair 1 is *S. pneumoniae* ST306/ST227 and pair 2 is *S. pneumoniae* ST611/ST618. Numbers indicate the number of genes.

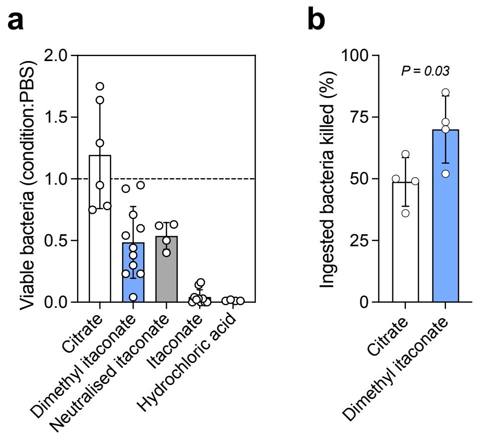

Supplementary Fig. 4: Effects of itaconate and dimethyl itaconate.
(A) *S. pneumoniae* D39 was incubated with citrate (5mM, pH 7.3), dimethyl itaconate (5mM, pH 6.9), neutralised itaconate (5mM, adjusted to pH 7.3), itaconate (5mM, pH 5.4), or hydrochloric acid (adjusted to pH 5.5). Bacterial viability after 1h, compared to PBS, is shown as a ratio. n=4-11 technical replicates. **(B)** Effect of 10mM exogenous dimethyl itaconate compared to citrate on hMDM early intracellular killing of *S. agalactiae* ST23 MOI=5 (n=4 biological replicates), compared using paired t-test. (A,B) Data presented as mean and standard deviation.

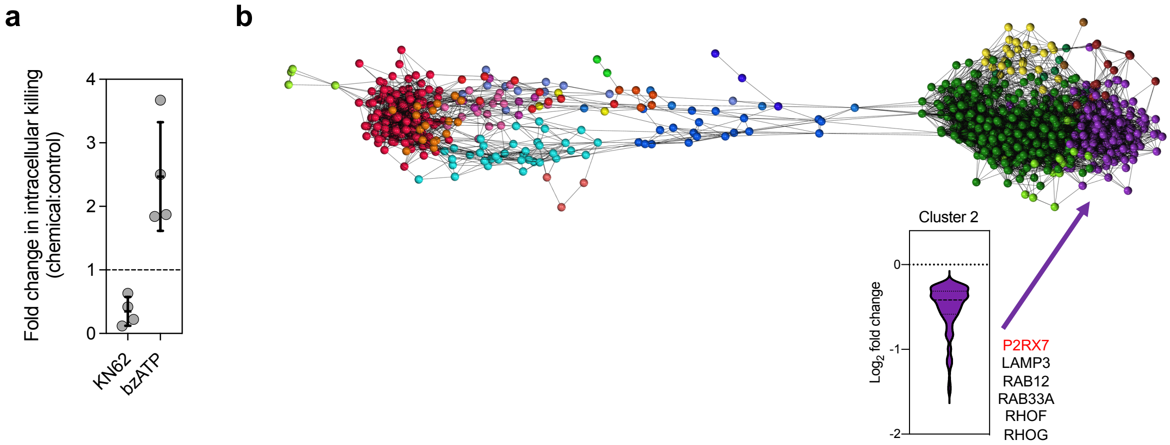

Supplementary Fig. 5: Macrophage P2RX7 signalling and gene expression.

(A) hMDM early intracellular killing of *S. pneumoniae* ST618 MOI=10 after treatment with 1μM KN62 or 3mM bzATP (n=4 biological replicates). Ratio <1 indicates a reduction in bacterial killing with chemical treatment and >1 indicates an increase. Data presented as mean and standard deviation. **(B)** Network analysis of hMDM gene co-expression for pair 2 (*S. pneumoniae* ST611 vs. ST618). Nodes represent differentially expressed genes (FDR<0.05), coloured by Markov Cluster Algorithm cluster membership (inflation value 1.7). Edges represent connections with a Pearson correlation coefficient ≥0.84. Log_2 fold change in the embedded violin plot represents expression in hMDM challenged with pneumococcal isolate escaping early intracellular killing (ST611) relative to the susceptible isolate (ST618).

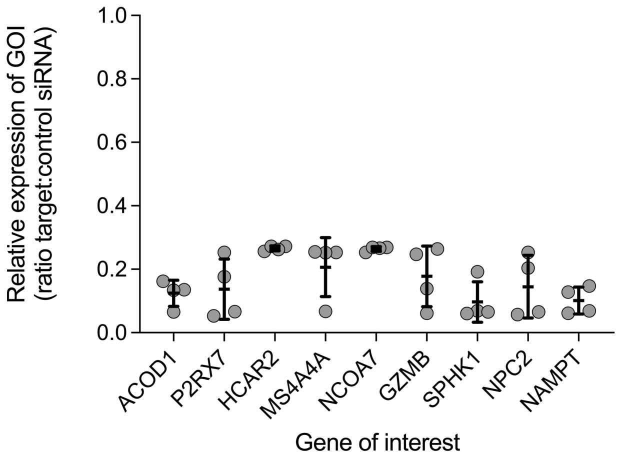

Supplementary Fig. 6: Effect of siRNA knockdown on transcript abundance.
Relative expression of individual genes expressed as a ratio of target:control siRNA transfected cells. A ratio of 1.0 indicates no reduction in transcript abundance whereas a ration <1 indicates a reduction associated with transfection of targeting siRNA. Relative gene expression calculated using the delta-delta Ct method with GAPDH as housekeeping gene. Data points represent biological replicates (n=4 per target). Bars represent mean and standard deviation. GOI: gene of interest.

Supplementary Table 1: Comparison of experimentally verified virulence factors

	CBPs	*PI-1*	*PI-2*	*PavA*	*PavB*	*PfbA*	*PspA*	*HysA*	*ZmpC*	*PsaA*	*LytA*	*NanA/B*	**Escape from killing**
ST306 3038	●	○	●	●	●	●	●	●	○	●	●	●	*
ST306 2627	●	○	●	●	●	●	○	●	○	●	●	●	*
ST617	●	○	●	●	●	●	○	●	○	●	●	●	*
ST227 INV104B	●	○	●	●	●	●	●	●	○	●	●	●	
ST227 S1-2	●	○	●	●	●	●	○	●	○	●	●	●	
ST217	●	○	○	●	●	●	●	●	●	●	●	●	
ST612	●	○	○	●	●	●	○	●	○	●	●	●	
ST618	●	○	○	●	●	●	●	●	●	●	●	●	
ST611	●	○	○	●	●	●	●	●	●	●	●	●	*
ST616	●	○	○	●	●	●	○	●	○	●	●	●	*
ST615	●	○	○	●	●	●	●	●	●	●	●	●	*

Sequences were uploaded to the BLAST using the Virulence Factors of Pathogenic Bacteria database(*25*). Presence or absence of known pneumococcal virulence factors (excluding pneumolysin) are presented. Red asterisk indicates the isolate was resistant to early intracellular killing by macrophages.

● virulence factor present; ○ virulence factor absent.

Supplementary Table 2: Clinical isolates of serotype 1 *Streptococcus pneumoniae* used

ST(*11*)	Isolate	Sample type	Diagnosis	Source/Reference
306	3038	Pleural fluid	Empyema	(*11*)
306	2627	Blood	Pneumonia	(*11*)
227	S1-2	Blood	Pneumonia	(*11*)
227	INV104B	Blood	Bacteraemia	(*11*)
617	S1-38	Blood	Pneumonia	(*11*)
217	S1-46	Blood	Unknown	(*11*)
618	S1-99	CSF	Meningitis	(*11*)
612	P1021	CSF	Meningitis	(*11*)
616	S1_102	CSF	Meningitis	(*11*)
615	NCTC 7465	Blood	Unknown	(*11*)
611	S1-71	CSF	Meningitis	(*11*)

ST: sequence type; CSF: cerebrospinal fluid.

Supplementary Table 3: Other bacterial isolates used

Species	Isolate	Source/Reference
Streptococcus pneumoniae	Serotype 14, NCTC 11902	NCTC
Streptococcus pneumoniae	Serotype 2 D39 wild type, NCTC 7466	(*81*)
Streptococcus pneumoniae	D39:*ply*-5	(*82*)
Streptococcus pneumoniae	D39 Δ*ply*	(*83*)
Streptococcus pneumoniae	D39 Δ*cps*	(*20*)
Streptococcus agalactiae	Serotype 1A, ST23, isolate "515"	(*84*)
Streptococcus agalactiae	Serotype 3, ST17, isolate "COH1"	(*85*)
Streptococcus agalactiae	Serotype 1a, ST17, isolate "A909"	(*86*)
Streptococcus pyogenes	$M1_{global}$ upper respiratory tract clinical isolate from 2012, "H1489"	(*56*)
Staphylococcus aureus	SH1000	(*87*)
Staphylococcus aureus	Newman	(*88*)
Staphylococcus aureus	USA300 LAC	(*89*)
Enterococcus faecium	VRE-EDI-160, ST262<br>*vanA* carrying clinical isolate (bacteraemia) with phenotypic resistance to amoxicillin and vancomycin, and high-level gentamicin resistance.	This study

ST: sequence type.

Supplementary Table 4: Studies included in MAIC

Ref.	Experimental category	Bacteria	Host	Cell/tissue	Model/disease
(*90*)	Transcriptomics	14	Human	AM	*In vitro* bacterial challenge
(*91*)	Transcriptomics	• 2 (D39)<br>• 19F (G54)<br>• 4 (TIGR4)	Human	Detroit 562 (epithelial)	*In vitro* bacterial adherence
(*92*)	Proteomics	2 (D39)	Human	hMDM	*In vitro* bacterial challenge
(*93*)	Transcriptomics	• 2 (D39)<br>• 4 (TIGR4)<br>• 6A	Mouse	• Nasopharyngeal tissue<br>• Lung tissue<br>• Blood	Pneumonia
(*94*)	Proteomics	4 (TIGR4)	Mouse	Lung tissue	Pneumonia
(*95*)	Transcriptomics	19 (ATCC 49619)	Mouse	• Lung tissue<br>• Neutrophils	Pneumonia
(*96*)	Transcriptomics	19 (ATCC 49619)	Mouse	Neutrophils	Pneumonia
(*97*)	Proteomics	Clinical	Human	CSF	Meningitis
(*98*)	Transcriptomics	2 (D39)	Human	hMDM	*In vitro* bacterial challenge
(*99*)	Proteomics	Clinical	Human	Blood	Pneumonia
(*100*)	Transcriptomics	2 (D39)	Mouse	Nasopharyngeal tissue	Carriage
(*101*)	Transcriptomics	Clinical	Human	Blood	Meningitis
(*102*)	Transcriptomics	3 (ATCC 6303)	Human	A549 (epithelial)	*In vitro* bacterial challenge
(*103*)	Transcriptomics	Clinical	Human	PBMC	Acute otitis media
(*104*)	Transcriptomics	1	Mouse	Lung tissue	Pneumonia
(*105*)	Transcriptomics	2 (D39)	Mouse	MH-S (AM)	*In vitro* bacterial challenge
(*106*)	Transcriptomics	14	Mouse	Lung tissue	Pneumonia
(*107*)	Transcriptomics	4	Human	hMDM	*In vitro* bacterial challenge
(*108*)	Transcriptomics	Clinical	Human	Blood	Pneumonia
(*109*)	Transcriptomics	3	Mouse	Lung tissue	Pneumonia
(*110*)	Transcriptomics	2 (D39)	Human	THP-1	*In vitro* bacterial challenge
(*111*)	Genetics	Clinical	Human	NA	Empyema
(*112*)	Transcriptomics	2 (D39)	Mouse	Lung tissue	Pneumonia
(*113*)	Transcriptomics	Clinical	Human	Monocytes	*In vitro* bacterial challenge
(*114*)	Proteomics	Clinical	Human	Blood	Pneumonia
(*115*)	Proteomics	Clinical	Human	CSF	Meningitis
(*116*)	Transcriptomics	2 (D39)	Mouse	• Blood<br>• Lung tissue	Invasive disease
(*117*)	Transcriptomics	• 6B<br>• 23F<br>• 4 (TIGR4)	Human	Detroit 562 (epithelial)	*In vitro* bacterial adherence
(*118*)	Transcriptomics	Unknown	Mouse	Spleen tissue	Pneumonia

AM: alveolar macrophage; hMDM: human monocyte derived macrophage; CSF: cerebrospinal fluid; PBMC: peripheral blood mononuclear cell

Supplementary Data Files

Supplementary Data File 1: RNAseq results for pair 1 comparison

Results of differential expression analysis of hMDM RNAseq, comparing transcriptional response to *S. pneumoniae* ST306 3038 vs. ST227 INV104B 4h after bacterial challenge.

Column names: "Gene": gene name; "logFC": $\log_2$ fold change; "FDR": false discovery rate.

Supplementary Data File 2: RNAseq results for pair 2 comparison

Results of differential expression analysis of hMDM RNAseq, comparing transcriptional response to *S. pneumoniae* ST611 vs. ST618 4h after bacterial challenge.

Column names: "Gene": gene name; "logFC": $\log_2$ fold change; "FDR": false discovery rate.

Supplementary Data File 3: MAIC output

Output of MAIC analysis of host factors involved in response to S. pneumoniae.

Column names: "gene": gene name; "maic_score": MAIC score

REFERENCES

1. GBD 2021 Antimicrobial Resistance Collaborators, Global burden of bacterial antimicrobial resistance 1990–2021: A systematic analysis with forecasts to 2050. *Lancet* **404**, 1199–1226 (2024).

2. Considering the host in host–pathogen interactions. *Nat. Microbiol.* **9**, 1149 (2024).

3. M. F. Barber, J. R. Fitzgerald, Mechanisms of host adaptation by bacterial pathogens. *FEMS Microbiol. Rev.* **48**, fuae019 (2024).

4. G. J. Wilson, K. S. Seo, R. A. Cartwright, T. Connelley, O. N. Chuang-Smith, J. A. Merriman, C. M. Guinane, J. Y. Park, G. A. Bohach, P. M. Schlievert, W. I. Morrison, J. R. Fitzgerald, A novel core genome-encoded superantigen contributes to lethality of community-associated MRSA necrotizing pneumonia. *PLOS Pathog.* **7**, e1002271 (2011).

5. A. W. Ensminger, Y. Yassin, A. Miron, R. R. Isberg, Experimental evolution of *Legionella pneumophila* in mouse macrophages leads to strains with altered determinants of environmental survival. *PLOS Pathog.* **8**, e1002731 (2012).

6. J. Alves, M. Vrieling, N. Ring, G. Yebra, A. Pickering, T. K. Prajsnar, S. A. Renshaw, J. R. Fitzgerald, Experimental evolution of *Staphylococcus aureus* in macrophages: Dissection of a conditional adaptive trait promoting intracellular survival. *mBio* **15**, e0034624 (2024).

7. C. Chaguza, M. Yang, L. C. Jacques, S. D. Bentley, A. Kadioglu, Serotype 1 pneumococcus: Epidemiology, genomics, and disease mechanisms. *Trends Microbiol.* **30**, 581–592 (2022).

8. E. Balsells, R. Dagan, I. Yildirim, P. P. Gounder, A. Steens, C. Muñoz-Almagro, C. Mameli, R. Kandasamy, N. Givon Lavi, L. Daprai, A. van der Ende, K. Trzciński, S. A. Nzenze, S. Meiring, D. Foster, L. R. Bulkow, K. Rudolph, A. Valero-Rello, S. Ducker, D. F. Vestrheim, A. von Gottberg, S. I. Pelton, G. Zuccotti, A. J. Pollard, E. A. M. Sanders, H. Campbell, S. A. Madhi, H. Nair, M. H. Kyaw, The relative invasive disease potential of *Streptococcus pneumoniae* among children after PCV introduction: A systematic review and meta-analysis. *J. Infect.* **77**, 368–378 (2018).

9. K. Sjöström, C. Spindler, A. Ortqvist, M. Kalin, A. Sandgren, S. Kühlmann-Berenzon, B. Henriques-Normark, Clonal and capsular types decide whether pneumococci will act as a primary or opportunistic pathogen. *Clin. Infect. Dis.* **42**, 451–459 (2006).

10. J. Leimkugel, A. Adams Forgor, S. Gagneux, V. Pflüger, C. Flierl, E. Awine, M. Naegeli, J. P. Dangy, T. Smith, A. Hodgson, G. Pluschke, An outbreak of serotype 1 *Streptococcus pneumoniae* meningitis in northern Ghana with features that are characteristic of *Neisseria meningitidis* meningitis epidemics. *J. Infect. Dis.* **192**, 192–199 (2005).

11. A. B. Brueggemann, B. G. Spratt, Geographic distribution and clonal diversity of *Streptococcus pneumoniae* serotype 1 isolates. *J. Clin. Microbiol.* **41**, 4966–4970 (2003).

12. D. H. Dockrell, H. M. Marriott, L. R. Prince, V. C. Ridger, P. G. Ince, P. G. Hellewell, M. K. B. Whyte, Alveolar macrophage apoptosis contributes to pneumococcal clearance in a resolving model of pulmonary infection. *J. Immunol.* **171**, 5380–5388 (2003).

13. D. Coggon, H. Inskip, P. Winter, B. Pannett, Lobar pneumonia: An occupational disease in welders. *Lancet* **344**, 41–43 (1994).

14. A. Wong, T. J. Marrie, S. Garg, J. D. Kellner, G. J. Tyrrell, SPAT Group, Welders are at increased risk for invasive pneumococcal disease. *Int. J. Infect. Dis.* **14**, e796–e799 (2010).

15. J. M. Antonini, N. J. Lawryk, G. G. Murthy, J. D. Brain, Effect of welding fume solubility on lung macrophage viability and function in vitro. *J. Toxicol. Environ. Health A* **58**, 343–363 (1999).

16. G. Ercoli, V. E. Fernandes, W. Y. Chung, J. J. Wanford, S. Thomson, C. D. Bayliss, K. Straatman, P. R. Crocker, A. Dennison, L. Martinez-Pomares, P. W. Andrew, E. R. Moxon, M. R. Oggioni, Intracellular replication of *Streptococcus pneumoniae* inside splenic macrophages serves as a reservoir for septicaemia. *Nat. Microbiol.* **3**, 600–610 (2018).

17. M. A. Bewley, J. A. Preston, M. Mohasin, H. M. Marriott, R. C. Budd, J. Swales, P. Collini, D. R. Greaves, R. W. Craig, C. E. Brightling, L. E. Donnelly, P. J. Barnes, D. Singh, S. D. Shapiro, M. K. B. Whyte, D. H. Dockrell, Impaired mitochondrial microbicidal responses in chronic

obstructive pulmonary disease macrophages. *Am. J. Respir. Crit. Care Med.* **196**, 845–855 (2017).

18. P. J. Collini, M. A. Bewley, M. Mohasin, H. M. Marriott, R. F. Miller, A. M. Geretti, A. Beloukas, A. Papadimitropoulos, R. C. Read, M. Noursadeghi, D. H. Dockrell, HIV gp120 in the lungs of antiretroviral therapy-treated individuals impairs alveolar macrophage responses to pneumococci. *Am. J. Respir. Crit. Care Med.* **197**, 1604–1615 (2018).

19. W. Nörenberg, C. Hempel, N. Urban, H. Sobottka, P. Illes, M. Schaefer, Clemastine potentiates the human P2X7 receptor by sensitizing it to lower ATP concentrations. *J. Biol. Chem.* **286**, 11067–11081 (2011).

20. T. K. Prajsnar, B. J. Michno, N. Pooranachandran, A. K. Fenton, T. J. Mitchell, D. H. Dockrell, S. A. Renshaw, Phagosomal acidification is required to kill *Streptococcus pneumoniae* in a zebrafish model. *Cell. Microbiol.* **2022**, 9429516 (2022).

21. J. A. Preston, M. A. Bewley, H. M. Marriott, A. McGarry Houghton, M. Mohasin, J. Jubrail, L. Morris, Y. L. Stephenson, S. Cross, D. R. Greaves, R. W. Craig, N. van Rooijen, C. D. Bingle, R. C. Read, T. J. Mitchell, M. K. B. Whyte, S. D. Shapiro, D. H. Dockrell, Alveolar macrophage apoptosis-associated bacterial killing helps prevent murine pneumonia. *Am. J. Respir. Crit. Care Med.* **200**, 84–97 (2019).

22. J. M. Jefferies, C. H. Johnston, L. A. Kirkham, G. J. Cowan, K. S. Ross, A. Smith, S. C. Clarke, A. B. Brueggemann, R. C. George, B. Pichon, G. Pluschke, V. Pfluger, T. J. Mitchell, Presence of nonhemolytic pneumolysin in serotypes of *Streptococcus pneumoniae* associated with disease outbreaks. *J. Infect. Dis.* **196**, 936–944 (2007).

23. L. A. Kirkham, J. M. Jefferies, A. R. Kerr, Y. Jing, S. C. Clarke, A. Smith, T. J. Mitchell, Identification of invasive serotype 1 pneumococcal isolates that express nonhemolytic pneumolysin. *J. Clin. Microbiol.* **44**, 151–159 (2006).

24. M. Witzenrath, F. Pache, D. Lorenz, U. Koppe, B. Gutbier, C. Tabeling, K. Reppe, K. Meixenberger, A. Dorhoi, J. Ma, A. Holmes, G. Trendelenburg, M. M. Heimesaat, S. Bereswill,

M. van der Linden, J. Tschopp, T. J. Mitchell, N. Suttorp, B. Opitz, The NLRP3 inflammasome is differentially activated by pneumolysin variants and contributes to host defense in pneumococcal pneumonia. *J. Immunol.* **187**, 434–440 (2011).

25. L. Chen, J. Yang, J. Yu, Z. Yao, L. Sun, Y. Shen, Q. Jin, VFDB: A reference database for bacterial virulence factors. *Nucleic Acids Res.* **33**, D325–D328 (2005).

26. A. M. Houghton, W. O. Hartzell, C. S. Robbins, F. X. Gomis-Rüth, S. D. Shapiro, Macrophage elastase kills bacteria within murine macrophages. *Nature* **460**, 637–641 (2009).

27. L. Verdot, G. Lalmanach, V. Vercruysse, S. Hartmann, R. Lucius, J. Hoebeke, F. Gauthier, B. Vray, Cystatins up-regulate nitric oxide release from interferon-gamma-activated mouse peritoneal macrophages. *J. Biol. Chem.* **271**, 28077–28081 (1996).

28. S. J. Yoon, D. H. Jo, S. H. Park, J. Y. Park, Y. K. Lee, M. S. Lee, J. K. Min, H. Jung, T. D. Kim, S. R. Yoon, S. W. Chung, J. H. Kim, I. Choi, Y. J. Park, Thioredoxin-interacting protein promotes phagosomal acidification upon exposure to *Escherichia coli* through inflammasome-mediated caspase-1 activation in macrophages. *Front. Immunol.* **10**, 2636 (2019).

29. M. J. Sweet, D. Ramnath, A. Singhal, R. Kapetanovic, Inducible antibacterial responses in macrophages. *Nat. Rev. Immunol.* **25**, 92–107 (2025).

30. B. Wang, A. Law, T. Regan, N. Parkinson, J. Cole, C. D. Russell, D. H. Dockrell, M. U. Gutmann, J. K. Baillie, Systematic comparison of ranking aggregation methods for gene lists in experimental results. *Bioinformatics* **38**, 4927–4933 (2022).

31. B. Li, S. M. Clohisey, B. S. Chia, B. Wang, A. Cui, T. Eisenhaure, L. D. Schweitzer, P. Hoover, N. J. Parkinson, A. Nachshon, N. Smith, T. Regan, D. Farr, M. U. Gutmann, S. I. Bukhari, A. Law, M. Sangesland, I. Gat-Viks, P. Digard, S. Vasudevan, D. Lingwood, D. H. Dockrell, J. G. Doench, J. K. Baillie, N. Hacohen, Genome-wide CRISPR screen identifies host dependency factors for influenza A virus infection. *Nat. Commun.* **11**, 164 (2020).

32. S. Shi, A. Blumenthal, C. M. Hickey, S. Gandotra, D. Levy, S. Ehrt, Expression of many immunologically important genes in *Mycobacterium tuberculosis*-infected macrophages is

independent of both TLR2 and TLR4 but dependent on IFN-αβ receptor and STAT1. *J. Immunol.* **175**, 3318–3328 (2005).

33. A. Michelucci, T. Cordes, J. Ghelfi, A. Pailot, N. Reiling, O. Goldmann, T. Binz, A. Wegner, A. Tallam, A. Rausell, M. Buttini, C. L. Linster, E. Medina, R. Balling, K. Hiller, Immune-responsive gene 1 protein links metabolism to immunity by catalyzing itaconic acid production. *Proc. Natl. Acad. Sci. U.S.A.* **110**, 7820–7825 (2013).

34. J. Naujoks, C. Tabeling, B. D. Dill, C. Hoffmann, A. S. Brown, M. Kunze, S. Kempa, A. Peter, H.-J. Mollenkopf, A. Dorhoi, O. Kershaw, A. D. Gruber, L. E. Sander, M. Witzenrath, S. Herold, A. Nerlich, A. C. Hocke, I. van Driel, N. Suttorp, S. Bedoui, H. Hilbi, M. Trost, B. Opitz, IFNs modify the proteome of *Legionella*-containing vacuoles and restrict infection via IRG1-derived itaconic acid. *PLOS Pathog.* **12**, e1005408 (2016).

35. A. Swain, M. Bambouskova, H. Kim, P. S. Andhey, D. Duncan, K. Auclair, V. Chubukov, D. M. Simons, T. P. Roddy, K. M. Stewart, M. N. Artyomov, Comparative evaluation of itaconate and its derivatives reveals divergent inflammasome and type I interferon regulation in macrophages. *Nat. Metab.* **2**, 594–602 (2020).

36. A. M. Cameron, A. Castoldi, D. E. Sanin, L. J. Flachsmann, C. S. Field, D. J. Puleston, R. L. Kyle, A. E. Patterson, F. Hässler, J. M. Buescher, B. Kelly, E. L. Pearce, E. J. Pearce, Inflammatory macrophage dependence on NAD^+ salvage is a consequence of reactive oxygen species-mediated DNA damage. *Nat. Immunol.* **20**, 420–432 (2019).

37. G. Wang, T. Han, D. Nijhawan, P. Theodoropoulos, J. Naidoo, S. Yadavalli, H. Mirzaei, A. A. Pieper, J. M. Ready, S. L. McKnight, P7C3 neuroprotective chemicals function by activating the rate-limiting enzyme in NAD salvage. *Cell* **158**, 1324–1334 (2014).

38. A. Y. Soare, T. L. Freeman, A. K. Min, H. S. Malik, E. O. Osota, T. H. Swartz, P2RX7 at the host-pathogen interface of infectious diseases. *Microbiol. Mol. Biol. Rev.* **85**, e00055-20 (2021).

39. I. P. Fairbairn, C. B. Stober, D. S. Kumararatne, D. A. Lammas, ATP-mediated killing of intracellular mycobacteria by macrophages is a $P2X_7$-dependent process inducing bacterial death by phagosome-lysosome fusion. *J. Immunol.* **167**, 3300–3307 (2001).

40. S. K. Garg, E. Volpe, G. Palmieri, M. Mattei, D. Galati, A. Martino, M. S. Piccioni, E. Valente, E. Bonanno, P. De Vito, P. M. Baldini, L. G. Spagnoli, V. Colizzi, M. Fraziano, Sphingosine 1–phosphate induces antimicrobial activity both in vitro and in vivo. *J. Infect. Dis.* **189**, 2129–2138 (2004).

41. M. Wheelwright, E. W. Kim, M. S. Inkeles, A. De Leon, M. Pellegrini, S. R. Krutzik, P. T. Liu, All-*trans* retinoic acid–triggered antimicrobial activity against *Mycobacterium tuberculosis* is dependent on NPC2. *J. Immunol.* **192**, 2280–2290 (2014).

42. V. Singh, S. Jamwal, R. Jain, P. Verma, R. Gokhale, K. V. S. Rao, *Mycobacterium tuberculosis*-driven targeted recalibration of macrophage lipid homeostasis promotes the foamy phenotype. *Cell Host Microbe* **12**, 669–681 (2012).

43. F. A. W. Verreck, T. de Boer, D. M. L. Langenberg, M. A. Hoeve, M. Kramer, E. Vaisberg, R. Kastelein, A. Kolk, R. de Waal-Malefyt, T. H. M. Ottenhoff, Human IL-23-producing type 1 macrophages promote but IL-10-producing type 2 macrophages subvert immunity to (myco)bacteria. *Proc. Natl. Acad. Sci. U.S.A.* **101**, 4560–4565 (2004).

44. J. Y. Jung, C. M. Robinson, IL-12 and IL-27 regulate the phagolysosomal pathway in mycobacteria-infected human macrophages. *Cell Commun. Signal* **12**, 16 (2014).

45. A. G. Rosas-Taraco, D. M. Higgins, J. Sánchez-Campillo, E. J. Lee, I. M. Orme, M. González-Juarrero, Intrapulmonary delivery of XCL1-targeting small interfering RNA in mice chronically infected with *Mycobacterium tuberculosis*. *Am. J. Respir. Cell Mol. Biol.* **41**, 136–145 (2009).

46. R. Custódio, C. J. McLean, A. E. Scott, J. Lowther, A. Kennedy, D. J. Clarke, D. J. Campopiano, M. Sarkar-Tyson, A. R. Brown, Characterization of secreted sphingosine-1-phosphate lyases required for virulence and intracellular survival of *Burkholderia pseudomallei*. *Mol. Microbiol.* **102**, 1004–1019 (2016).

47. J. K. Brieland, D. G. Remick, M. L. LeGendre, N. C. Engleberg, J. C. Fantone, In vivo regulation of replicative *Legionella pneumophila* lung infection by endogenous interleukin-12. *Infect. Immun.* **66**, 65–69 (1998).

48. M. Walch, F. Dotiwala, S. Mulik, J. Thiery, T. Kirchhausen, C. Clayberger, A. M. Krensky, D. Martinvalet, J. Lieberman, Cytotoxic cells kill intracellular bacteria through granulysin-mediated delivery of granzymes. *Cell* **157**, 1309–1323 (2014).

49. B. P. Howden, S. G. Giulieri, T. Wong Fok Lung, S. L. Baines, L. K. Sharkey, J. Y. H. Lee, A. Hachani, I. R. Monk, T. P. Stinear, *Staphylococcus aureus* host interactions and adaptation. *Nat. Rev. Microbiol.* **21**, 380–395 (2023).

50. A. R. Richardson, G. A. Somerville, A. L. Sonenshein, Regulating the intersection of metabolism and pathogenesis in gram-positive bacteria. *Microbiol. Spectr.* **3**, 10.1128/microbiolspec.MBP-0004-2014 (2015).

51. L. A. O'Neill, R. J. Kishton, J. Rathmell, A guide to immunometabolism for immunologists. *Nat. Rev. Immunol.* **16**, 553–565 (2016).

52. G. Krejčová, A. Danielová, P. Nedbalová, M. Kazek, L. Strych, G. Chawla, J. M. Tennessen, J. Lieskovská, M. Jindra, T. Doležal, A. Bajgar, *Drosophila* macrophages switch to aerobic glycolysis to mount effective antibacterial defense. *eLife* **8**, e50414 (2019).

53. M. G. Machado, T. A. Patente, Y. Rouillé, S. Heumel, E. M. Melo, L. Deruyter, B. Pourcet, V. Sencio, M. M. Teixeira, F. Trottein, Acetate improves the killing of *Streptococcus pneumoniae* by alveolar macrophages via NLRP3 inflammasome and glycolysis-HIF-1α axis. *Front. Immunol.* **13**, 773261 (2022).

54. K. Watson, C. D. Russell, J. K. Baillie, K. Dhaliwal, J. R. Fitzgerald, T. J. Mitchell, A. J. Simpson, S. A. Renshaw, D. H. Dockrell, Developing novel host-based therapies targeting microbicidal responses in macrophages and neutrophils to combat bacterial antimicrobial resistance. *Front. Immunol.* **11**, 786 (2020).

55. M. Souli, F. Ruffin, S. H. Choi, L. P. Park, S. Gao, N. C. Lent, B. K. Sharma-Kuinkel, J. T. Thaden, S. A. Maskarinec, L. Wanda, J. Hill-Rorie, B. Warren, B. Hansen, V. G. Fowler Jr., Changing characteristics of *Staphylococcus aureus* bacteremia: Results from a 21-year, prospective, longitudinal study. *Clin. Infect. Dis.* **69**, 1868–1877 (2019).

56. N. N. Lynskey, E. Jauneikaite, H. K. Li, X. Zhi, C. E. Turner, M. Mosavie, M. Pearson, M. Asai, L. Lobkowicz, J. Y. Chow, J. Parkhill, T. Lamagni, V. J. Chalker, S. Sriskandan, Emergence of dominant toxigenic M1T1 *Streptococcus pyogenes* clone during increased scarlet fever activity in England: A population-based molecular epidemiological study. *Lancet Infect. Dis.* **19**, 1209–1218 (2019).

57. D. Girlich, S. Ouzani, C. Emeraud, L. Gauthier, R. A. Bonnin, N. Le Sache, M. Mokhtari, I. Langlois, C. Begasse, N. Arangia, S. Fournier, N. Fortineau, T. Naas, L. Dortet, Uncovering the novel *Enterobacter cloacae* complex species responsible for septic shock deaths in newborns: A cohort study. *Lancet Microbe* **2**, e536–e544 (2021).

58. C. T. Fang, Y. P. Chuang, C. T. Shun, S. C. Chang, J. T. Wang, A novel virulence gene in *Klebsiella pneumoniae* strains causing primary liver abscess and septic metastatic complications. *J. Exp. Med.* **199**, 697–705 (2004).

59. M. Ebrahimi-Rad, P. Bifani, C. Martin, K. Kremer, S. Samper, J. Rauzier, B. Kreiswirth, J. Blazquez, M. Jouan, D. van Soolingen, B. Gicquel, Mutations in putative mutator genes of *Mycobacterium tuberculosis* strains of the W-Beijing family. *Emerg. Infect. Dis.* **9**, 838–845 (2003).

60. S. J. Jenkins, J. E. Allen, The expanding world of tissue-resident macrophages. *Eur. J. Immunol.* **51**, 1882–1896 (2021).

61. E. M. Ryan, P. Sadiku, P. Coelho, E. R. Watts, A. Zhang, A. J. M. Howden, M. A. Sanchez-Garcia, M. Bewley, J. Cole, B. J. McHugh, W. Vermaelen, B. Ghesquiere, P. Carmeliet, G. Rodriguez Blanco, A. Von Kriegsheim, Y. Sanchez, W. Rumsey, J. F. Callahan, G. Cooper, N. Parkinson, K. Baillie, D. A. Cantrell, J. McCafferty, G. Choudhury, D. Singh, D. H. Dockrell, M. K. B. Whyte, S. R. Walmsley, NRF2 activation reprograms defects in oxidative metabolism to

restore macrophage function in chronic obstructive pulmonary disease. *Am. J. Respir. Crit. Care Med.* **207**, 998–1011 (2023).

62. International Chronic Granulomatous Disease Cooperative Study Group, A controlled trial of interferon gamma to prevent infection in chronic granulomatous disease. *N. Engl. J. Med.* **324**, 509–516 (1991).

63. A. A. Miles, S. S. Misra, J. O. Irwin, The estimation of the bactericidal power of the blood. *J. Hyg.* **38**, 732–749 (1938).

64. A. Bankevich, S. Nurk, D. Antipov, A. A. Gurevich, M. Dvorkin, A. S. Kulikov, V. M. Lesin, S. I. Nikolenko, S. Pham, A. D. Prjibelski, A. V. Pyshkin, A. V. Sirotkin, N. Vyahhi, G. Tesler, M. A. Alekseyev, P. A. Pevzner, SPAdes: A new genome assembly algorithm and its applications to single-cell sequencing. *J. Comput. Biol.* **19**, 455–477 (2012).

65. A. M. Bolger, M. Lohse, B. Usadel, Trimmomatic: A flexible trimmer for Illumina sequence data. *Bioinformatics* **30**, 2114–2120 (2014).

66. T. J. Treangen, B. D. Ondov, S. Koren, A. M. Phillippy, The Harvest suite for rapid core-genome alignment and visualization of thousands of intraspecific microbial genomes. *Genome Biol.* **15**, 524 (2014).

67. T. Seemann, Prokka: Rapid prokaryotic genome annotation. *Bioinformatics* **30**, 2068–2069 (2014).

68. N. J. Croucher, A. J. Page, T. R. Connor, A. J. Delaney, J. A. Keane, S. D. Bentley, J. Parkhill, S. R. Harris, Rapid phylogenetic analysis of large samples of recombinant bacterial whole genome sequences using Gubbins. *Nucleic Acids Res.* **43**, e15 (2015).

69. A. J. Page, B. Taylor, A. J. Delaney, J. Soares, T. Seemann, J. A. Keane, S. R. Harris, *SNP-sites*: Rapid efficient extraction of SNPs from multi-FASTA alignments. *Microb. Genom.* **2**, e000056 (2016).

70. M. N. Price, P. S. Dehal, A. P. Arkin, FastTree: Computing large minimum evolution trees with profiles instead of a distance matrix. *Mol. Biol. Evol.* **26**, 1641–1650 (2009).

71. A. McColl, S. Bournazos, S. Franz, M. Perretti, B. P. Morgan, C. Haslett, I. Dransfield, Glucocorticoids induce protein S-dependent phagocytosis of apoptotic neutrophils by human macrophages. *J. Immunol.* **183**, 2167–2175 (2009).

72. D. H. Dockrell, M. Lee, D. H. Lynch, R. C. Read, Immune-mediated phagocytosis and killing of *Streptococcus pneumoniae* are associated with direct and bystander macrophage apoptosis. *J. Infect. Dis.* **184**, 713–722 (2001).

73. R. Patro, G. Duggal, M. I. Love, R. A. Irizarry, C. Kingsford, Salmon provides fast and bias-aware quantification of transcript expression. *Nat. Methods* **14**, 417–419 (2017).

74. M. D. Robinson, D. J. McCarthy, G. K. Smyth, edgeR: A Bioconductor package for differential expression analysis of digital gene expression data. *Bioinformatics* **26**, 139–140 (2010).

75. G. Korotkevich, V. Sukhov, N. Budin, B. Shpak, M. N. Artyomov, A. Sergushichev, Fast gene set enrichment analysis. bioRxiv 060012 [Preprint] (2021); https://doi.org/10.1101/060012.

76. N. Parkinson, N. Rodgers, M. Head Fourman, B. Wang, M. Zechner, M. C. Swets, J. E. Millar, A. Law, C. D. Russell, J. K. Baillie, S. Clohisey, Dynamic data-driven meta-analysis for prioritisation of host genes implicated in COVID-19. *Sci. Rep.* **10**, 22303 (2020).

77. L. S. Frost, A. Dhingra, J. Reyes-Reveles, K. Boesze-Battaglia, The use of DQ-BSA to monitor the turnover of autophagy-associated cargo. *Methods Enzymol.* **587**, 43–54 (2017).

78. W. J. Su, T. Zhang, C. L. Jiang, W. Wang, Clemastine alleviates depressive-like behavior through reversing the imbalance of microglia-related pro-inflammatory state in mouse hippocampus. *Front. Cell. Neurosci.* **12**, 412 (2018).

79. J. I. Lee, J. W. Park, K. J. Lee, D. H. Lee, Clemastine improves electrophysiologic and histomorphometric changes through promoting myelin repair in a murine model of compression neuropathy. *Sci. Rep.* **11**, 20886 (2021).

80. C. Nüsslein-Volhard, R. Dahm, Eds., *Zebrafish: A Practical Approach* (Oxford Univ. Press, 2002).

81. J. A. Lanie, W. L. Ng, K. M. Kazmierczak, T. M. Andrzejewski, T. M. Davidsen, K. J. Wayne, H. Tettelin, J. I. Glass, M. E. Winkler, Genome sequence of Avery's virulent serotype 2 strain D39 of *Streptococcus pneumoniae* and comparison with that of unencapsulated laboratory strain R6. *J. Bacteriol.* **189**, 38–51 (2007).

82. D. C. Badgujar, A. Anil, A. E. Green, M. V. Surve, S. Madhavan, A. Beckett, I. A. Prior, B. K. Godsora, S. B. Patil, P. K. More, S. G. Sarkar, A. Mitchell, R. Banerjee, P. S. Phale, T. J. Mitchell, D. R. Neill, P. Bhaumik, A. Banerjee, Structural insights into loss of function of a pore forming toxin and its role in pneumococcal adaptation to an intracellular lifestyle. *PLOS Pathog.* **16**, e1009016 (2020).

83. D. Fatykhova, A. Rabes, C. Machnik, K. Guruprasad, F. Pache, J. Berg, M. Toennies, T. T. Bauer, P. Schneider, M. Schimek, S. Eggeling, T. J. Mitchell, A. M. Mitchell, R. Hilker, T. Hain, N. Suttorp, S. Hippenstiel, A. C. Hocke, B. Opitz, Serotype 1 and 8 pneumococci evade sensing by inflammasomes in human lung tissue. *PLOS ONE* **10**, e0137108 (2015).

84. C. J. Baker, M. S. Edwards, B. J. Webb, D. L. Kasper, Antibody-independent classical pathway-mediated opsonophagocytosis of type Ia, group B streptococcus. *J. Clin. Invest.* **69**, 394–404 (1982).

85. N. Vollmuth, T. Sauerwein, K. U. Foerstner, A. J. Westermann, A. Schubert-Unkmeir, D. W. Lam, B. J. Kim, *Streptococcus agalactiae* strain COH1 transcriptome in association with stem cell-derived brain-like endothelial cells. *Microbiol. Resour. Announc.* **13**, e0045524 (2024).

86. H. Tettelin, V. Masignani, M. J. Cieslewicz, C. Donati, D. Medini, N. L. Ward, S. V. Angiuoli, J. Crabtree, A. L. Jones, A. S. Durkin, R. T. DeBoy, T. M. Davidsen, M. Mora, M. Scarselli, I. Margarit y Ros, J. D. Peterson, C. R. Hauser, J. P. Sundaram, W. C. Nelson, R. Madupu, L. M. Brinkac, R. J. Dodson, M. J. Rosovitz, S. A. Sullivan, S. C. Daugherty, D. H. Haft, J. Selengut, M. L. Gwinn, L. Zhou, N. Zafar, H. Khouri, D. Radune, G. Dimitrov, K. Watkins, K. J. B. O'Connor, S. Smith, T. R. Utterback, O. White, C. E. Rubens, G. Grandi, L. C. Madoff, D. L.

Kasper, J. L. Telford, M. R. Wessels, R. Rappuoli, C. M. Fraser, Genome analysis of multiple pathogenic isolates of *Streptococcus agalactiae*: Implications for the microbial “pan-genome”. *Proc. Natl. Acad. Sci. U.S.A.* **102**, 13950–13955 (2005).

87. A. J. O'Neill, *Staphylococcus aureus* SH1000 and 8325–4: Comparative genome sequences of key laboratory strains in staphylococcal research. *Lett. Appl. Microbiol.* **51**, 358–361 (2010).

88. E. S. Duthie, L. L. Lorenz, Staphylococcal coagulase: Mode of action and antigenicity. *J. Gen. Microbiol.* **6**, 95–107 (1952).

89. L. K. McDougal, C. D. Steward, G. E. Killgore, J. M. Chaitram, S. K. McAllister, F. C. Tenover, Pulsed-field gel electrophoresis typing of oxacillin-resistant *Staphylococcus aureus* isolates from the United States: Establishing a national database. *J. Clin. Microbiol.* **41**, 5113–5120 (2003).

90. M. A. Bewley, R. C. Budd, E. Ryan, J. Cole, P. Collini, J. Marshall, U. Kolsum, G. Beech, R. D. Emes, I. Tcherniaeva, G. A. M. Berbers, S. R. Walmsley, G. Donaldson, J. A. Wedzicha, I. Kilty, W. Rumsey, Y. Sanchez, C. E. Brightling, L. E. Donnelly, P. J. Barnes, D. Singh, M. K. B. Whyte, D. H. Dockrell, COPDMAP, Opsonic phagocytosis in chronic obstructive pulmonary disease is enhanced by Nrf2 agonists. *Am. J. Respir. Crit. Care Med.* **198**, 739–750 (2018).

91. H. J. Bootsma, M. Egmont-Petersen, P. W. Hermans, Analysis of the in vitro transcriptional response of human pharyngeal epithelial cells to adherent *Streptococcus pneumoniae*: Evidence for a distinct response to encapsulated strains. *Infect. Immun.* **75**, 5489–5499 (2007).

92. J. Cole, A. Angyal, R. D. Emes, T. J. Mitchell, M. J. Dickman, D. H. Dockrell, Pneumolysin is responsible for differential gene expression and modifications in the epigenetic landscape of primary monocyte derived macrophages. *Front. Immunol.* **12**, 573266 (2021).

93. A. D'Mello, A. N. Riegler, E. Martínez, S. M. Beno, T. D. Ricketts, E. F. Foxman, C. J. Orihuela, H. Tettelin, An in vivo atlas of host-pathogen transcriptomes during *Streptococcus pneumoniae* colonization and disease. *Proc. Natl. Acad. Sci. U.S.A.* **117**, 33507–33518 (2020).

94. M. Ferrer-Navarro, A. Strehlitz, E. Medina, J. Vila, Changed expression of cytoskeleton proteins during lung injury in a mouse model of *Streptococcus pneumoniae* infection. *Front. Microbiol.* **9**, 928 (2018).

95. J. C. Gomez, H. Dang, J. R. Martin, C. M. Doerschuk, Nrf2 modulates host defense during *Streptococcus pneumoniae* pneumonia in mice. *J. Immunol.* **197**, 2864–2879 (2016).

96. J. C. Gomez, H. Dang, M. Kanke, R. S. Hagan, J. R. Mock, S. N. P. Kelada, P. Sethupathy, C. M. Doerschuk, Predicted effects of observed changes in the mRNA and microRNA transcriptome of lung neutrophils during *S. pneumoniae* pneumonia in mice. *Sci. Rep.* **7**, 11258 (2017).

97. G. Gómez-Baena, R. J. Bennett, C. Martínez-Rodríguez, M. Wnęk, G. Laing, G. Hickey, L. McLean, R. J. Beynon, E. D. Carrol, Quantitative proteomics of cerebrospinal fluid in paediatric pneumococcal meningitis. *Sci. Rep.* **7**, 7042 (2017).

98. K. Griss, W. Bertrams, A. Sittka-Stark, K. Seidel, C. Stielow, S. Hippenstiel, N. Suttorp, M. Eberhardt, J. Wilhelm, J. Vera, B. Schmeck, MicroRNAs constitute a negative feedback loop in *Streptococcus pneumoniae*-induced macrophage activation. *J. Infect. Dis.* **214**, 288–299 (2016).

99. H. Huang, R. C. Ideh, E. Gitau, M. L. Thézénas, M. Jallow, B. Ebruke, O. Chimah, C. Oluwalana, H. Karanja, G. Mackenzie, R. A. Adegbola, D. Kwiatkowski, B. M. Kessler, J. A. Berkley, S. R. Howie, C. Casals-Pascual, Discovery and validation of biomarkers to guide clinical management of pneumonia in African children. *Clin. Infect. Dis.* **58**, 1707–1715 (2014).

100. E. A. Joyce, S. J. Popper, S. Falkow, *Streptococcus pneumoniae* nasopharyngeal colonization induces type I interferons and interferon-induced gene expression. *BMC Genomics* **10**, 404 (2009).

101. B. W. Kulohoma, F. Marriage, O. Vasieva, L. Mankhambo, K. Nguyen, M. E. Molyneux, E. M. Molyneux, P. J. R. Day, E. D. Carrol, Peripheral blood RNA gene expression in children with pneumococcal meningitis: A prospective case-control study. *BMJ Paediatr. Open* **1**, e000092 (2017).

102. H. Li, L. Lin, L. Chong, S. Gu, S. Wen, G. Yu, X. Hu, L. Dong, H. Zhang, C. Li, Time-resolved mRNA and miRNA expression profiling reveals crucial coregulation of molecular pathways involved in epithelial-pneumococcal interactions. *Immunol. Cell Biol.* **98**, 726–742 (2020).

103. K. Liu, L. Chen, R. Kaur, M. Pichichero, Transcriptome signature in young children with acute otitis media due to *Streptococcus pneumoniae*. *Microbes Infect.* **14**, 600–609 (2012).

104. J. M. Marqués, A. Rial, N. Muñoz, F. X. Pellay, L. Van Maele, H. Léger, T. Camou, J. C. Sirard, A. Benecke, J. A. Chabalgoity, Protection against *Streptococcus pneumoniae* serotype 1 acute infection shows a signature of Th17- and IFN-γ-mediated immunity. *Immunobiology* **217**, 420–429 (2012).

105. I. Martínez, J. C. Oliveros, I. Cuesta, J. de la Barrera, V. Ausina, C. Casals, A. de Lorenzo, E. García, B. García-Fojeda, J. Garmendia, M. González-Nicolau, A. Lacoma, M. Menéndez, D. Moranta, A. Nieto, J. Ortín, A. Pérez-González, C. Prat, E. Ramos-Sevillano, V. Regueiro, A. Rodriguez-Frandsen, D. Solís, J. Yuste, J. A. Bengoechea, J. A. Melero, Apoptosis, Toll-like, RIG-I-like and NOD-like receptors are pathways jointly induced by diverse respiratory bacterial and viral pathogens. *Front. Microbiol.* **8**, 276 (2017).

106. V. Minhas, R. Aprianto, L. J. McAllister, H. Wang, S. C. David, K. T. McLean, I. Comerford, S. R. McColl, J. C. Paton, J. W. Veening, C. Trappetti, In vivo dual RNA-seq reveals that neutrophil recruitment underlies differential tissue tropism of *Streptococcus pneumoniae*. *Commun. Biol.* **3**, 293 (2020).

107. J. Periselneris, G. Ercoli, T. Pollard, S. Chimalapati, E. Camberlein, G. Szylar, C. Hyams, G. Tomlinson, F. C. Petersen, R. A. Floto, M. Noursadeghi, J. S. Brown, Relative contributions of extracellular and internalized bacteria to early macrophage proinflammatory responses to *Streptococcus pneumoniae*. *mBio* **10**, e02144-19 (2019).

108. O. Ramilo, W. Allman, W. Chung, A. Mejias, M. Ardura, C. Glaser, K. M. Wittkowski, B. Piqueras, J. Banchereau, A. K. Palucka, D. Chaussabel, Gene expression patterns in blood leukocytes discriminate patients with acute infections. *Blood* **109**, 2066–2077 (2007).

109. N. D. Ritchie, T. J. Evans, Dual RNA-seq in *Streptococcus pneumoniae* infection reveals compartmentalized neutrophil responses in lung and pleural space. *mSystems* **4**, e00216-19 (2019).

110. P. D. Rogers, J. Thornton, K. S. Barker, D. O. McDaniel, G. S. Sacks, E. Swiatlo, L. S. McDaniel, Pneumolysin-dependent and -independent gene expression identified by cDNA microarray analysis of THP-1 human mononuclear cells stimulated by *Streptococcus pneumoniae*. *Infect. Immun.* **71**, 2087–2094 (2003).

111. A. Salas, J. Pardo-Seco, R. Barral-Arca, M. Cebey-López, A. Gómez-Carballa, I. Rivero-Calle, S. Pischedda, M. J. Currás-Tuala, J. Amigo, J. Gómez-Rial, F. Martinón-Torres, GENDRES Network, Whole exome sequencing identifies new host genomic susceptibility factors in empyema caused by *Streptococcus pneumoniae* in children: A pilot study. *Genes* **9**, 240 (2018).

112. B. P. Scicluna, M. H. van Lieshout, D. C. Blok, S. Florquin, T. van der Poll, Modular transcriptional networks of the host pulmonary response during early and late pneumococcal pneumonia. *Mol. Med.* **21**, 430–441 (2015).

113. S. Tchatalbachev, R. Ghai, H. Hossain, T. Chakraborty, Gram-positive pathogenic bacteria induce a common early response in human monocytes. *BMC Microbiol.* **10**, 275 (2010).

114. M. H. Tsai, T. Y. Lin, S. Y. Hsieh, C. Y. Chiu, C. H. Chiu, Y. C. Huang, Comparative proteomic studies of plasma from children with pneumococcal pneumonia. *Scand. J. Infect. Dis.* **41**, 416–424 (2009).

115. E. C. Wall, P. Brownridge, G. Laing, V. S. Terra, V. Mlozowa, B. Denis, M. Nyirenda, T. Allain, E. Ramos-Sevillano, E. Carrol, A. Collins, S. B. Gordon, D. G. Lalloo, B. Wren, R. Beynon, R. S. Heyderman, J. S. Brown, CSF levels of elongation factor Tu is associated with increased mortality in Malawian adults with *Streptococcus pneumoniae* meningitis. *Front. Cell. Infect. Microbiol.* **10**, 603623 (2020).

116. M. Weber, S. Lambeck, N. Ding, S. Henken, M. Kohl, H. P. Deigner, D. P. Enot, E. I. Igwe, L. Frappart, M. Kiehntopf, R. A. Claus, T. Kamradt, D. Weih, Y. Vodovotz, D. E. Briles, A. D.

Ogunniyi, J. C. Paton, U. A. Maus, M. Bauer, Hepatic induction of cholesterol biosynthesis reflects a remote adaptive response to pneumococcal pneumonia. *FASEB J.* **26**, 2424–2436 (2012).

117. C. M. Weight, C. Venturini, S. Pojar, S. P. Jochems, J. Reiné, E. Nikolaou, C. Solórzano, M. Noursadeghi, J. S. Brown, D. M. Ferreira, R. S. Heyderman, Microinvasion by *Streptococcus pneumoniae* induces epithelial innate immunity during colonisation at the human mucosal surface. *Nat. Commun.* **10**, 3060 (2019).

118. H. Zhang, Y. A. Su, P. Hu, J. Yang, B. Zheng, P. Wu, J. Peng, Y. Tang, L. Zhang, Signature patterns revealed by microarray analyses of mice infected with influenza virus A and *Streptococcus pneumoniae*. *Microbes Infect.* **8**, 2172–2185 (2006).
